# Endogenous Methanol Regulates Mammalian Gene Activity

**DOI:** 10.1371/journal.pone.0090239

**Published:** 2014-02-27

**Authors:** Tatiana V. Komarova, Igor V. Petrunia, Anastasia V. Shindyapina, Denis N. Silachev, Ekaterina V. Sheshukova, Gleb I. Kiryanov, Yuri L. Dorokhov

**Affiliations:** 1 A. N. Belozersky Institute of Physico-Chemical Biology, Moscow State University, Moscow, Russia; 2 N. I. Vavilov Institute of General Genetics, Russian Academy of Science, Moscow, Russia; University of Saarland Medical School, Germany

## Abstract

We recently showed that methanol emitted by wounded plants might function as a signaling molecule for plant-to-plant and plant-to-animal communications. In mammals, methanol is considered a poison because the enzyme alcohol dehydrogenase (ADH) converts methanol into toxic formaldehyde. However, the detection of methanol in the blood and exhaled air of healthy volunteers suggests that methanol may be a chemical with specific functions rather than a metabolic waste product. Using a genome-wide analysis of the mouse brain, we demonstrated that an increase in blood methanol concentration led to a change in the accumulation of mRNAs from genes primarily involved in detoxification processes and regulation of the alcohol/aldehyde dehydrogenases gene cluster. To test the role of ADH in the maintenance of low methanol concentration in the plasma, we used the specific ADH inhibitor 4-methylpyrazole (4-MP) and showed that intraperitoneal administration of 4-MP resulted in a significant increase in the plasma methanol, ethanol and formaldehyde concentrations. Removal of the intestine significantly decreased the rate of methanol addition to the plasma and suggested that the gut flora may be involved in the endogenous production of methanol. ADH in the liver was identified as the main enzyme for metabolizing methanol because an increase in the methanol and ethanol contents in the liver homogenate was observed after 4-MP administration into the portal vein. Liver mRNA quantification showed changes in the accumulation of mRNAs from genes involved in cell signalling and detoxification processes. We hypothesized that endogenous methanol acts as a regulator of homeostasis by controlling the mRNA synthesis.

## Introduction

In higher plants, gaseous methanol has traditionally been considered a biochemical “waste product” [1]. However, the effects of pectin methylesterase (PME)-generated plant methanol (“emitters”) on plant defensive reactions (“receivers”) were recently studied [2]. These investigations demonstrated that increased methanol emissions from PME-transgenic or mechanically wounded non-transgenic plants retarded the growth of the bacterial pathogen *Ralstonia solanacearum* in neighboring “receiver” plants. This antibacterial resistance was accompanied by an upregulation of genes that control stress and cell-to-cell communication in the “receiver.” These results suggest that methanol is a signaling molecule for within-plant and plant-to-plant communications

In humans, methanol is considered a poison because alcohol dehydrogenase (ADH) metabolizes methanol into toxic formaldehyde [3], [4]. A second, three-step metabolism reaction then occurs. In the first step, formaldehyde is oxidized to *S*-formylglutathione in a reaction that requires reduced glutathione and is mediated by a NAD-dependent formaldehyde dehydrogenase or alcohol dehydrogenase 3 (ADH3) [5]. In the next step, thiolase catalyzes the conversion of *S*-formylglutathione to formic acid, which dissociates to form formate and a hydrogen ion. The third reaction (formate to CO_2_ and water) is catalyzed by catalase through a combination with tetrahydrofolate to produce 10-formyl tetrahydrofolate [6]. The semitransformation of formaldehyde to formic acid takes 1–2 minutes in many species, including humans [7]. Formaldehyde did not accumulate substantially in methanol–intoxicated humans [6]–[8]. Moreover, formaldehyde was not detected in blood, urine, or tissues obtained from animals treated with methanol, and humans poisoned with this alcohol had no increase in formaldehyde [4].

Interestingly, the breath of healthy people contains a small amount of methanol [9]. Recent data have confirmed that methanol and short-lived formaldehyde are natural compounds found in normal, healthy human individuals [7], [10], [11]. Methanol has been identified in the exhaled breath of healthy volunteers [11]. The methanol content in human blood increases after the ingestion of plant food associated with the activity of the PME, which is capable of generating methanol during the fermentation of pectin [12]. The level of methanol in the blood of healthy volunteers was 1000 times lower than the level measured in postmortem specimens from methanol fatalities [8].

The origin of endogenous methanol in humans has not yet been elucidated, but two sources have been suggested [9]: anaerobic fermentation by gut bacteria and certain metabolic processes that transform *S*-adenosyl methionine into methanol [13]. Recently, a significant elevation of endogenous ethanol and methanol was found in the plasma of healthy women and men after intake of the specific ADH inhibitor 4-methylpyrazole (4-MP) [14], [15]. This finding indicates that a high level of methanol is generated by endogenous sources in humans. These data suggest that methanol is either a metabolic waste product or a chemical with specific functions in humans. To better understand the role of methanol in mammalian metabolism, we have recently identified methanol-sensitive genes [12]. We used HeLa cells lacking ADH [16] to eliminate any confounding effects from genes that are involved in formaldehyde and formic acid detoxification on the analysis. We showed that mice preferred the odor of methanol to the odors of other plant volatiles. Moreover, inhaled methanol altered the accumulation of mRNA in the mouse brain. From this finding, we concluded that methanol may have a role in plant-animal and intra-animal signaling.

In this study, a genome-wide analysis suggested that methanol is involved in the regulation of cellular processes in the mouse brain, such as the up- and down-regulation of genes involved in methanol detoxification and ADH regulation. We used 4-MP to show that there was a high capacity for methanol production from endogenous sources in mice. The ADH suppression resulted in a significant increase in the level of plasma methanol and also in the contents of ethanol and formaldehyde. Removal of the intestine significantly decreased the rate of methanol addition to the plasma and suggested that the gut flora may be involved in the endogenous production of methanol. The liver is likely the main organ for methanol metabolism. After ADH suppression and the subsequent endogenous methanol increase, we found a change in the accumulation of mRNA in the liver from genes involved in methanol regulation.

## Results

### Genome-wide analysis of the mouse brain after methanol administration

**Figure 1:**
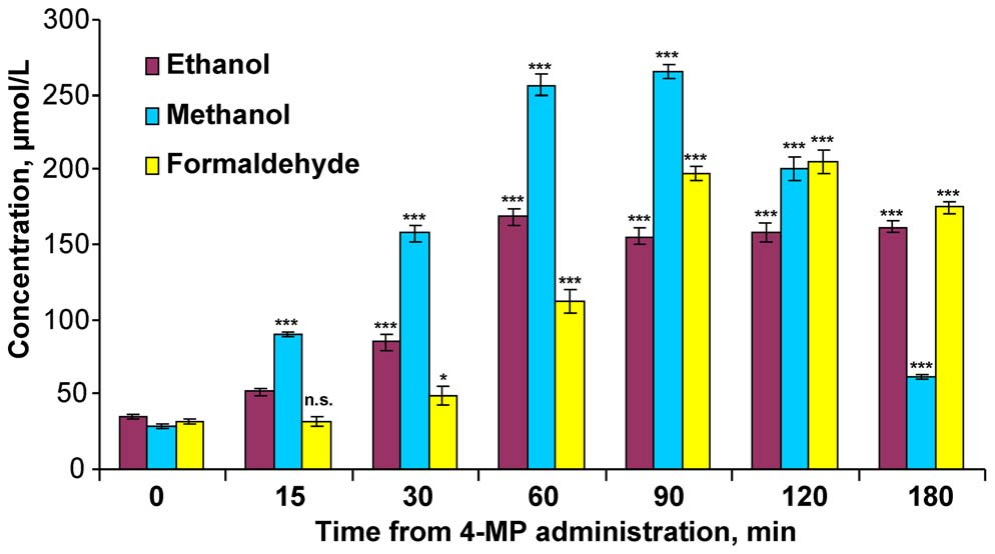
Dynamics of methanol, formaldehyde and ethanol changes in the blood plasma of mice after 4-MP administration. Each mouse in the treatment and control groups received an intraperitoneal injection of 4-MP (10 mg/kg) or the saline solution, respectively. Blood samples were analyzed for methanol/ethanol and formaldehyde content by GC and HPLC analyses, respectively. The data are shown with standard error bars, and *P*-values (Student's *t*-test) are designated by: ***, *P*<0.001; *, *P*<0.05; n.s., not significant.

Previously, we demonstrated the existence of methanol-sensitive genes, which are preferentially activated in the mouse brain after an increase in blood methanol levels [12]. This result suggests that there is genetic control of methanol metabolism. Therefore, we studied the effect of methanol on cellular responses in the murine brain. Methanol was administered to one group of mice by intraperitoneal injection (0.12 g/kg). This treatment resulted in a sharp increase in the blood methanol content 60 min post-injection and a subsequent decrease in the methanol content after 180 min (Table S1). Another group of mice was treated with 4-methylpyrazole (4-MP) to suppress ADH. We determined that 4-MP treatment led to the rapid accumulation of methanol in the blood. Figure 1 shows that 15 min after intraperitoneal administration of 4-MP, the methanol concentration in the blood increased dramatically and continued to rise for over 90 min. After 90 min, the methanol level decreased until the 180 min time point. The level of formaldehyde in the blood mirrored the kinetics of methanol but with a 30 min delay. The ethanol level increased at the same rate as that of methanol but did not show a considerable drop by 180 min. These data showed that there is a substantial source of methanol in the mouse, which is clearly observed after the 4-MP-mediated inhibition of ADH.

**Figure 2:**
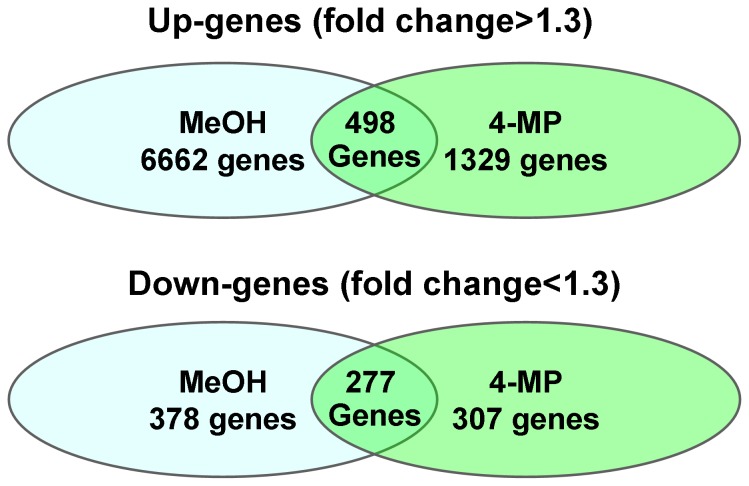
Microarray analysis of differentially regulated murine brain mRNAs after 4-MP or methanol administration. Venn diagram of the genes that are differentially expressed after 4-MP and methanol administration compared to the control mice after saline solution injection. Genes were analyzed using the J-Express gene expression analysis software. The number of genes commonly regulated is indicated by the intersection of the circles. All the genes included in this analysis had significant changes in their expression compared to the control, with a *P*-value <0.05.

**Figure 3:**
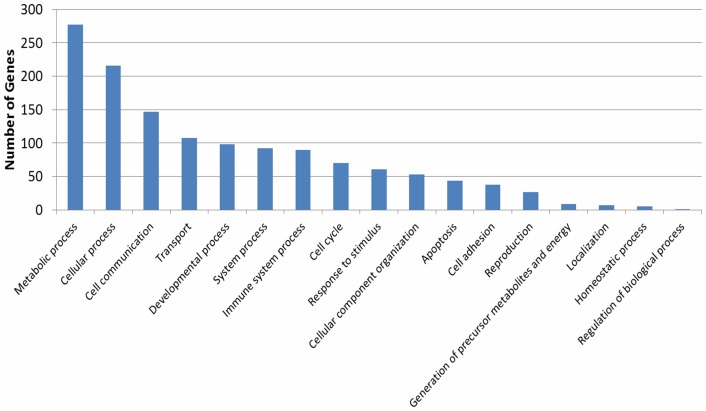
Diagram of biological processes with differentially expressed genes after 4-MP and methanol administration. Genes significantly differentially expressed in treated and control mice were analyzed using the Gene Ontology (GO) tool in the PANTHER database to categorize the biological processes in which they participate.

Next, RNA samples collected from the brain after methanol and 4-MP treatment were used to generate cDNAs. These cDNAs were analyzed using Illumina Whole-Genome 6 microarrays with probes for approximately 48,200 transcripts. We identified the transcripts that were expressed with detection *P*-values of <0.05. Figure 2 shows a Venn diagram of the genes that were differentially expressed after 4-MP and methanol administration compared with the control injection of saline solution. The number and list of genes (Table S2 and S3) that were regulated by both methanol and 4-MP administration are shown in the intersection of the circles (Fig. 2). The biological processes that were highly represented in the BLASTx GO analyses included metabolic, cellular, cell communication and transport processes (Fig. 3). The list of significantly regulated *Mus musculus* genes involved in the processes of detoxification, oxidative phosphorylation, cell signaling and beta-amyloid metabolism included 18 up-regulated and 6 down-regulated genes (Table 1).

**Table 1:** List of significantly up- and down-regulated *Mus musculus* genes in mouse brains after 4-MP administration.

Gene symbol	Gene description	Genome location	Accession number	*q*-value	Fold change vs. control	Gene function
						(from KEGG*)
**Upregulated genes**						
RUSC2#	RUN and SH3 domain containing 2 protein	4qA5	NM_199057.2	<0.001	2.071	Cell signaling
GSTP1	Glutathione S-transferase, pi 1	19qA	NM_013541.1	<0.001	1.782	Detoxification
MGST3	Microsomal glutathione S-transferase 3	1qH2.3	NM_025569.1	<0.001	1.694	Detoxification
PSENEN	Presenilin enhancer 2 homolog *(C. elegans*)	7qB1	NM_025498.2	<0.001	1.688	Component of the gamma-secretase protein complex required for intramembranous processing of the beta-amyloid precursor
PTGDS	Prostaglandin D2 synthase 2, hematopoietic	6qC1	NM_008963.1	<0.001	1.670	Neuromodulator and trophic factor in the central nervous system
NDUFA6	NADH dehydrogenase 1 alpha subcomplex, 6	15qE1	NM_025987.1	<0.001	1.619	Oxidative phosphorylation and involvement in Alzheimer's and Huntington's diseases
NDUFB9	NADH dehydrogenase 1 beta subcomplex, 9	15qD1	NM_023172.3	<0.001	1.565	Oxidative phosphorylation and involvement in Alzheimer's and Huntington's diseases
SLC6A3	Solute carrier family 6 (neurotransmitter transporter, dopamine), member 3 (Slc6a3)	13qC1	NM_010020.3	<0.001	1.551	The dopamine transporter; involvement in Parkinson's disease
NDUFA5	NADH dehydrogenase 1 alpha subcomplex, 5	6qA3.1	NM_026614.2	<0.001	1.547	Oxidative phosphorylation and involvement in Alzheimer's and Huntington's diseases
SRXN1#	Sulfiredoxin 1 homolog (S. cerevisiae)	2qG3	NM_029688.2	<0.001	1.515	Contributes to oxidative stress resistance
NDUFB6	NADH dehydrogenase 1 beta subcomplex, 6	4qA5	NM_001033305.1	<0.001	1.506	Oxidative phosphorylation and involvement in Alzheimer's and Huntington's diseases
SESN1	Sestrin 1	10qB2	NM_001013370.1	<0.001	1.503	Antioxidant function
PARK7	Parkinson disease (autosomal recessive, early onset) 7	4qE2	NM_020569.1	<0.001	1.447	Antioxidant function and involvement in Parkinson's disease
SNCA	Synuclein, alpha, transcript variant 2	6qB3	NM_009221.2	<0.001	1.445	Component of amyloid plaques in the brains of patients with Alzheimer's disease.
GSTO1	Glutathione S-transferase omega 1	19qD1	NM_010362.2	<0.001	1.438	Detoxification
PRDX1	Peroxiredoxin 1	4qD1	NM_011034.4	<0.001	1.390	Antioxidant function
CYP2D22	Cytochrome P450, family 2, subfamily d, polypeptide 26	15qE1	NM_019823.3	<0.001	1.347	Dopamine biosynthetic process, involvement in Parkinson's disease
NDUFC2	NADH dehydrogenase (ubiquinone) 1, subcomplex unknown, 2	7qE1	NM_024220.1	<0.001	1.325	Oxidative phosphorylation and involvement in Alzheimer's and Huntington's diseases
ALDH2#	Aldehyde dehydrogenase 2, mitochondrial	5qF	NM_009656.3	<0.001	1.290	Responsible for breaking down acetaldehyde and formaldehyde produced by ethanol and methanol, respectively
**Downregulated genes**						
CPLX2#	Complexin 2	13qB1	NM_009946.2	<0.001	−2.604	Cell signaling
FIBCD1#	Fibrinogen C domain containing 1	2qB	NM_178887.3	<0.001	−1.956	Cell signaling
ATP2A3	ATPase, Ca++ transporting, ubiquitous.	11qB4	NM_016745.2	<0.001	−1.492	Involvement in ion channel transport and Alzheimer's disease
NCAM1	Neural cell adhesion molecule 1, transcript variant 1	9qA5.3	NM_010875.3	<0.001	−1.443	Involvement in cell-to-cell interactions
SERPINA3H#	Serine (or cysteine) peptidase inhibitor, clade A, member 3H	12qE	NM_001034870.2	<0.001	−1.420	Response to cytokine stimulus
APOE#	Apolipoprotein E	7qA3	NM_009696.2	<0.001	−1.419	Involvement in cholesterol metabolism and genetic risk factor for late-onset sporadic Alzheimer disease
GNAQ	Guanine nucleotide binding protein, alpha q polypeptide	19qA	NM_008139.5	0.027	−1.377	Modulator or transducer in various transmembrane signaling systems
TESK1#	Testis specific protein kinase 1	4qB1	NM_011571.2	<0.001	−1.302	Cell signaling
MT-ATP6	ATP synthase subunit a	Chromosome MT: 7,927–8,607	BC012020.1	<0.001	−1.300	Respiratory electron transport

*KEGG, Kyoto Encyclopedia of Genes and Genomes (http://www.genome.jp/kegg/).

# Genes selected for further analysis.

### Verification of microarray data with qRT-PCR

**Figure 4:**
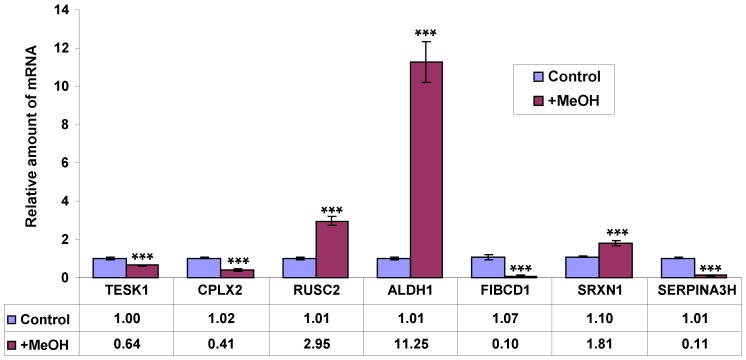
Verification of microarray data with qRT-PCR. Murine brain mRNAs were quantified by qRT-PCR after treatment with methanol by inhalation. The data shown represent five independent experiments. ***, *P*<0.001 (Student's *t*-test).

**Figure 5:**
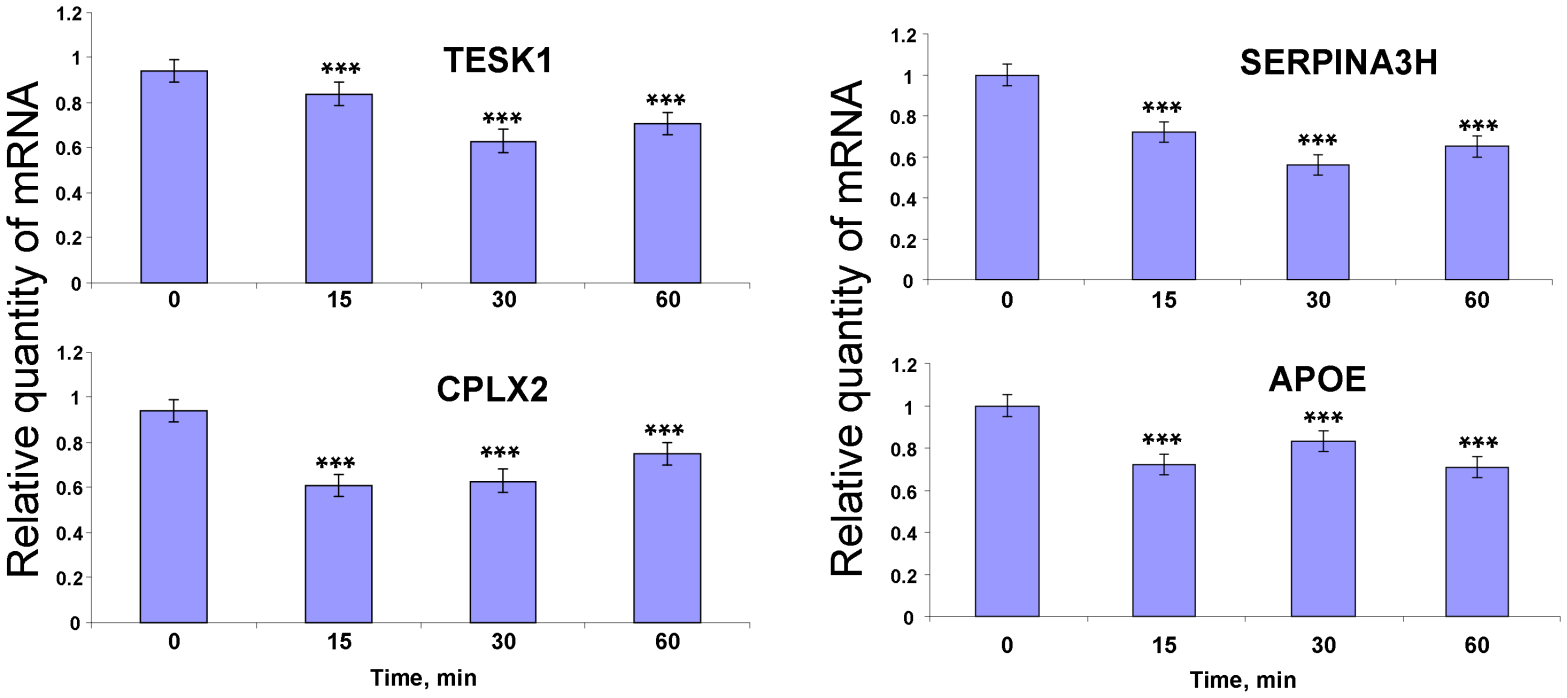
qRT-PCR analysis of murine brain mRNAs content after intraperitoneal 4-MP administration. The data shown represent five independent experiments. ***, *P*<0.001 (Student's *t*-test).

Oligonucleotide primers for the 8 genes related to cell signaling, oxidative stress resistance and detoxification were used for validation of the microarray data by qRT-PCR. Verification of the microarray data was conducted using two experimental approaches. In the first approach, the mice were treated with methanol by inhalation (Fig. 4). In the second approach, the mice were injected intraperitoneally with 4-MP (Fig. 5). The results for all 8 tested genes agreed with the findings of the microarray assay (Table 1). Interestingly, we detected dynamic changes in the transcript abundance of genes 1 hour after 4-MP treatment.

### The gastrointestinal tract is involved in the formation of endogenous methanol in the blood plasma of mammals

**Figure 6:**
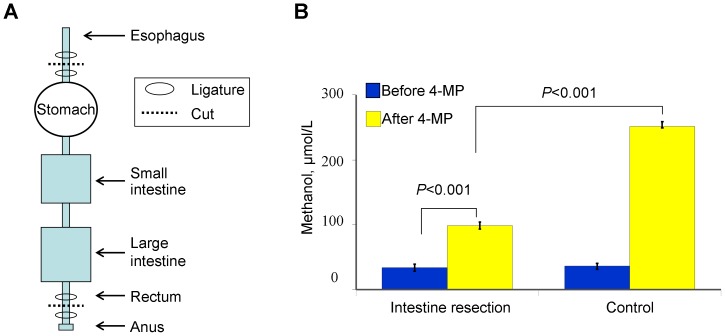
The examination of the putative role of intestinal microbes in the generation of methanol in rats. **A** - Scheme of the rat gastrointestinal tract resection. **B** - The diagram showing levels of methanol in the blood of rats before and after resection of the gastrointestinal tract. The data are shown with standard error bars, and *P*-values (Student's *t*-test) are indicated.

Although the origin of endogenous methanol in humans and mammals is not known, anaerobic metabolism by gut bacteria is a putative methanol source [9]. We tested whether the intestinal microbes in rats generate methanol. If the intestine is the site of methanol synthesis, then the blood methanol concentration of rats in which the bowel has been removed will significantly differ from the blood methanol concentration in the control animals after administration of 4-MP. Figure 6 shows that the removal of the bowel results in the lower increase of methanol in the blood caused by the administration of 4-MP compared to the control group.

**Figure 7:**
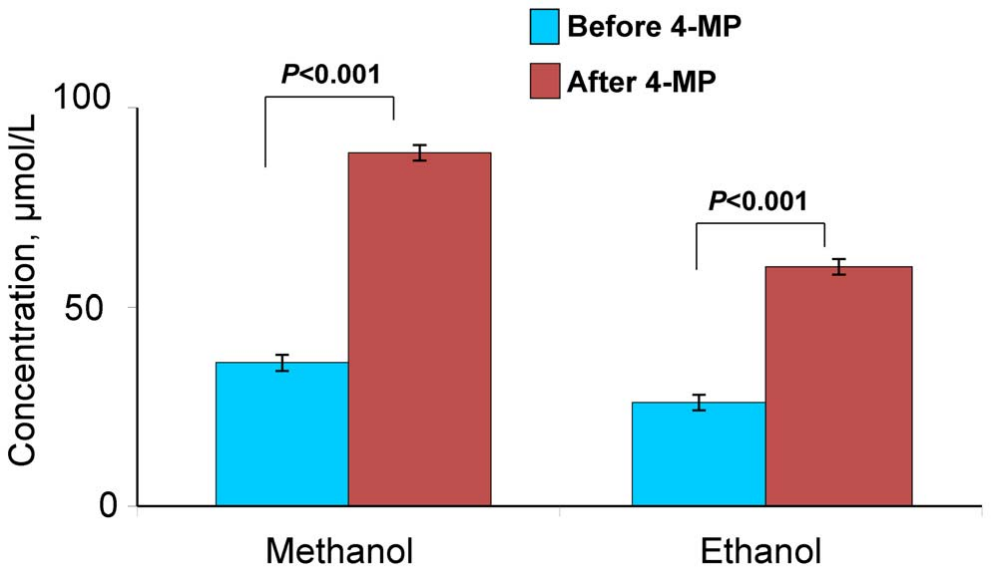
Rat liver ADH suppression results in an increase of endogenous methanol and ethanol. Measurements of liver methanol and ethanol content 30-MP (10 mg/kg) administration directly into the portal vein. The data are shown with standard error bars, and *P*-values (Student's *t*-test) are indicated.

We hypothesized that the methanol generated by the intestinal flora passes through the portal vein into the liver. Once in the liver, the methanol can undergo primary metabolism with ADH. To test this hypothesis, we injected 4-MP into the portal vein and measured the methanol content in the liver. Figure 7 shows that there was a sharp increase in the content of methanol and ethanol in liver homogenates 30 min after 4-MP administration into the portal vein.

We concluded that the gastrointestinal tract of the rat is the primary site of methanol formation and of its subsequent fermentation.

### Quantification of liver mRNA after ADH inhibition

**Figure 8:**
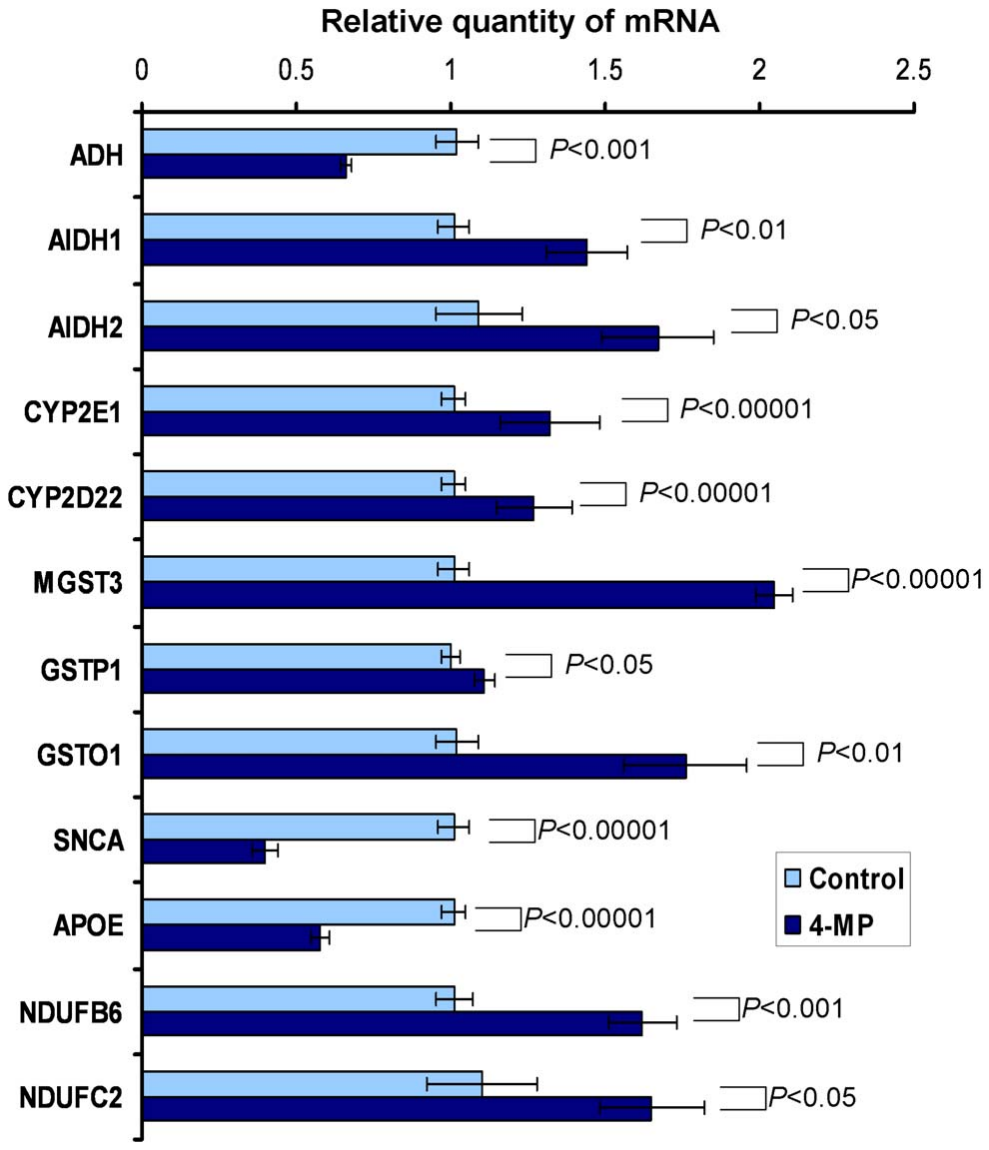
The suppression of mouse liver ADH results in the accumulation of mRNA of the ADH/AlDH gene cluster. The liver mRNA was quantified by qRT-PCR after 4-MP (10 mg/kg) administration. The data are shown with standard error bars, and *P*-values (Student's *t*-test) are indicated.

We hypothesized that the methanol generated by the intestinal flora causes the activation and mobilization of genes involved in methanol metabolism in liver cells. To test this hypothesis, we quantified the mRNA in the liver to identify genes that we had previously identified in our genome-wide analysis of the mouse brain (Table 1). Mice were injected with 4-MP, liver samples were collected and mRNA content was measured using qRT-PCR. Figure 8 shows that the inhibition of ADH activity led to an appreciable reduction in the mRNA level of the ADH gene. At the same time, there was an increase in the amount of liver aldehyde dehydrogenase (AlDH1 and AlDH2) mRNA and other mRNAs of this cluster, such as cytochrome P450 (CYP2E1, CYP2D22) and glutathione S-transferases (MGST3, GSTP1 and GSTO1). The quantity of SNCA and APOE mRNA was significantly reduced in the liver after ADH inhibition. However, the mRNAs of the NADH dehydrogenases genes (NDUFB6, NDUFC2), which are involved in the processes of oxidative phosphorylation, were increased.

We concluded that the suppression of ADH activity, which leads to a sharp increase in blood methanol, stimulates the transcription of genes involved in detoxifying processes.

## Discussion

Methanol and short-lived formaldehyde are natural compounds in normal, healthy individuals [9]–[12]. A significant elevation of endogenous ethanol and methanol in the plasma of healthy women and men following administration of the specific ADH inhibitor 4-MP [15] indicates that humans generate a high level of endogenous methanol. We have shown that administration of 4-MP to mice leads to a rapid increase in the blood content of methanol and ethanol (Fig. 1). Unexpectedly, after a short delay, there was also a substantial increase in the formaldehyde levels. Until now, it was believed that the half-life of formaldehyde was too short to allow it to be detected in animals [4]. However, our experiments have shown that the inhibition of ADH by 4-MP is accompanied by a significant increase in formaldehyde (Fig. 1). As hypothesized, the intestinal microflora is responsible for the formation of methanol in the intestine. However, the bacterial species responsible for this process have not yet been identified or isolated. We have shown that in rats, the gastrointestinal tract is directly involved in the production of endogenous methanol. The removal of the bowel lowered the increase in the methanol content in the blood after rats were administered 4-MP (Fig. 6). We believe that the significant increase in methanol in the blood of animals after treatment with 4-MP is caused by the inhibition of the liver ADH rather than the ADH of the intestinal microflora. This conclusion is supported by two considerations. First, in our experiments, 4-MP was administered by intraperitoneal injection, which bypasses the gastrointestinal tract, and this led to an increase in blood methanol 15 min after injection (Fig. 1). Second, the direct administration of 4-MP into the portal vein led to a higher content of methanol and ethanol in the liver homogenate (Fig. 7).

These data, however, do not exclude the existence of other metabolic processes that could lead to the formation of methanol. Our experiments showed that the removal of the bowel significantly reduced, but did not eliminate, the increase in blood methanol after administration of 4-MP (Fig. 6). Protein carboxylmethylation involves the methylation of amino acid COOH groups. This reaction is catalyzed by methyltransferases and results in the production of carboxyl methyl esters that are readily hydrolyzed in neutral and basic pH conditions by methylesterase to produce methanol [17]. Protein carboxymethylase is highly localized in the brain and pituitary gland of several mammalian species [18]. Interestingly, aspartame, which is a widely used synthetic non-nutritive sweetener, is a methyl ester of a dipeptide (*N*-*L*-*α*-aspartyl-*L*-phenylalanine) that is likely converted to methanol with the participation of protein methylesterases [7].

The results of this study suggest that methanol may be not a metabolic waste product but rather a chemical with specific functions in mammals. Although methanol, similar to carbon dioxide, may be exhaled into the air as a waste product, this chemical may also be a molecule that can induce biochemical processes important for cellular function. To test this hypothesis, we conducted a genome-wide analysis in mice with high blood methanol levels and found increases in the brain mRNA transcripts involved in detoxification processes (Table 1). Although the origin of endogenous methanol in humans and mammals is unclear, methanol is an important factor that is regulated by the ADH gene cluster.

Information about the affected proteins was obtained by analyzing the potential functional associations between the identified proteins using the STRING 9.0 server, which is a database that provides information about known and predicted protein-protein interactions. This analysis revealed 3 consistent groups of functional associations (Fig. S1). The first functional interaction group consists of two glutathione S-transferases (GSTP1 and MGST3), mitochondrial aldehyde dehydrogenase 2 (AlDH2) and Cytochrome P450 2E1 (CYP2e1), which is an important enzyme that converts ethanol to acetaldehyde and acetate in humans. The second functional association group includes proteins involved in cholesterol metabolism (APOE) and amyloid plaque formation (SNCA). The third group is composed of proteins involved in oxidative phosphorylation, Alzheimer's and Huntington's diseases (NADH dehydrogenases and PSENEN), antioxidant function and Parkinson's disease (PARK7).

Protein-protein interactions predicted by the STRING 9.0 server (Fig. S1) require confirmation. We showed that the suppression of the ADH activity leads to a sharp increase in blood methanol and stimulates the activity of genes involved in detoxifying processes (Fig. 8). We therefore hypothesized that the increase of blood methanol levels, such as from plant food intake, leads to the activation of genes that contribute to the metabolism of methanol as part of a feedback loop to return to normal levels. This process may be regulated by the SNCA and APOE genes, which are involved in cholesterol metabolism and the pathogenesis of Alzheimer's disease. The function of these genes in the regulation of ADH and AlDH is unclear; however, endogenous methanol and formaldehyde can be important factors in the pathogenesis of human neurodegenerative diseases. This hypothesis is confirmed by several lines of evidence. First, progressive senile dementia is accompanied by increased production of endogenous formaldehyde [19]. Second, AlDH inhibition can lead to Parkinson's disease [20]. To assess whether methanol is helpful or detrimental to humans, we need to keep in mind the positive role of fruits and raw vegetables in human health. A vegetarian diet is the main source of exogenous methanol for a healthy person [12], [21]. The role of methanol-generating pectin in atherosclerosis and cancer prophylaxis is well known [22]. Pectin can modulate detoxifying enzymes, stimulate the immune system, modulate cholesterol synthesis and act as an antibacterial, antioxidant or neuroprotective agent [23].

## Materials and Methods

### Animal experiments

Experiments were performed on outbred white male rats and BALB/c mice. The animals had unlimited access to food and water and were kept in cages with a temperature controlled environment (20±1°C) with the lights on from 9 AM to 9 PM. The care of experimental animals was performed in strict accordance with the guidelines of the “Euroguide on the accommodation and care of animals used for experimental and other scientific purposes” (FELASA, 2007). All experimental protocols were approved by the Animal Ethics Committees of the A. N. Belozersky Institute of Physico-Chemical Biology, Moscow State University, Moscow, Russia (Protocol Registration number 2/12 of 6^th^ February 2012). Euthanasia was performed using carbon dioxide in accordance with the 2000 Report of the AVMA Panel on Euthanasia, and all efforts were made to minimize animal suffering. For all surgical procedures, rats were anesthetized by intraperitoneal injection of 300 mg/kg (12%) chloral hydrate. Additionally, to ensure proper pain relief in the preoperative and postoperative periods, we used repeated topical application of a long-acting local anesthetic bupivacaine ointment. Moribund animals, animals obviously in pain, or animals showing signs of severe and enduring distress were humanely killed. Criteria for making the decision to kill moribund or severely suffering animals, and guidance on the recognition of predictable or impending death, were performed in accordance with the Guidelines for Endpoints in Animal Study Proposals (DHHS NIH Office of Animal Care and Use).

### Mouse brain microarrays

BALB/c mice were randomly divided into groups of five mice. The mice were injected intraperitoneally with methanol (0.12 g/kg) or 4-MP (10 mg/kg), and two hours later, brain samples were collected after mice decapitation. The mice injected intraperitoneally with saline solution were used as a control. Whole brains homogenates from biological replicates were subjected to RNA isolation using TRIzol (Invitrogen, USA), according to the manufacturer's instructions. Following isolation, total RNA was purified and concentrated using the RNeasy MinElute Kit (QIAGEN, Hilden, Germany). Total RNA (400 ng) was prepared for microarray using the Illumina TotalPrep RNA Amplification Kit (Ambion, USA). The brain transcriptome was assessed using Illumina MouseRef-6 BeadChip microarrays, which contain 45,200 specific oligonucleotide probes. Arrays were scanned using the Illumina BeadArray Reader and BeadScan software. Data were analyzed using GenomeStudio v.2012 (Illumina, USA) with normalization by Cubic Spline and differential expression analysis using the Illumina Custom algorithm. This analysis generated a list of probes with significant (*P*<0.05) differences in signal intensity between treated and control mice. Probe annotations from the microarray manifest file were updated using the SOURCE database (http://smd.stanford.edu/cgi-bin/source/sourceSearch) with the listed NCBI transcript accession numbers as the search terms. In cases where the accession number was no longer listed in the database, annotations were updated by aligning the probe sequence against the mouse transcriptome using BLAST (http://blast.ncbi.nlm.nih.gov/Blast.cgi). Genes were analyzed using the J-Express gene expression analysis software, SAM (Significance Analysis of Microarrays) tool.

### Mouse experiments with 4-MP administration

BALB/c mice were randomly divided into groups of ten mice. Each mouse in the treatment group directly received intraperitoneal introduction of 4-MP (10 mg/kg). In the control group the saline solution was introduced intraperitoneally. After 15, 30, 60, 90, 120 or 180 min, blood samples (50 µl) were isolated from the tail vein of the mice. Samples were incubated at 4°C for 2 h for cell sedimentation, and then an equal volume of 10% trichloroacetic acid (TCA) was added to the plasma. The mixture was incubated for 20 min on ice and then centrifuged for 10 min at 16 000 g. Finally, the supernatant was analyzed for methanol/ethanol and formaldehyde content by GC and HPLC analyses, respectively.

### Rat intestine resection and 4-MP administration via portal vein

Rats randomly divided into groups of five rats. The rats were anesthetized with a 12% solution of chloral hydrate at a dose of 300 mg/kg. A sample of 1 ml of blood was collected from the jugular vein, and an operation was carried out to remove the intestine (esophagus from the stomach, small and large intestine). The animals were injected intravenously with 1.5 ml of 4-MP (10 mg/kg) and maintained for 1 hour under heat lamps connected to a thermostat to maintain body temperature at a physiological level. After 1 hour, the animals were bled again from the jugular vein. Animals with a peritoneum cut without removal of intestine were used as a control. To determine the influence of 4-MP and methanol metabolism in the liver, 1 ml of a solution of 4-MP (10 mg/kg) was administered in the portal vein of rats. Control rats were injected with 1 ml of physiological saline. After 30 min, liver samples (300 mg) were homogenized in 300 µl PBS buffer. After centrifugation, the supernatant was collected and methanol content was detected.

### Methanol and ethanol measurements by gas chromatography

Methanol and ethanol content was determined by gas chromatography on a capillary FFAP column (50 m×0.32 mm; Varian Inc., Lake Forest, CA, USA) in a Kristall 2000 gas chromatograph (Eridan, Russia). Liquid samples were measured under the following operating conditions: carrier gas, nitrogen; nitrogen flow, 30 ml/min; air flow, 400 ml/min; hydrogen flow, 40 ml/min; injected volume, 1 µl; injector temperature, 160°C; column temperature, 75°C, increased at 15°C/min to 150°C; retention time, 6.5 min (methanol) or 6.43 min (ethanol); and flame ionization detector temperature, 240°C.

### Formaldehyde measurements by HPLC system

The HPLC system Dionex Ultimate 3000 consisting of a four-gradient pump, degasser mobile phase, automatic injector (autosampler) combined with column thermostat, and a spectrophotometric detector with a variable wavelength detector (with simultaneous detection according to 4 different wavelengths) was used. The chromatographic column was Synergi Hydro-RP, 250 mm×4.6, with a grain diameter of sorbent 4 microns (porosity 80 Å) and a precolumn Security Guard (C18 cartridge, diameter 3 mm) (Phenomenex, USA). The stationary phase was silica grafted with polar groups C18 endcapped. The mobile phase was a mixture of deionized water and acetonitrile (HPLC grade) in a 50/50 ratio by volume. The mobile phase flow rate was 1 ml/min, the column temperature control was set at 30°C, and the sample injection volume was 20 µl of a pre-washing injector mixture of acetonitrile and water (50/50) and the sample. The total analysis time was 20 minutes. Detection at the spectrophotometer flow cell was at 360 nm (near-UV). Determination of formaldehyde was based on its interaction with an excess of 2,4-dinitrophenylhydrazine in an acidic medium to form the corresponding hydrazone colored product, which was separated by chromatography from the remaining components of the solution. To obtain a reagent solution, 100 µl of 85% phosphoric acid was added to 20 ml of pure acetonitrile, and then a 20 mg sample of 2,4-dinitrophenylhydrazone hydrochloride was added. To prepare the blank solution, 0.5 ml of deionized water was added to 0.5 ml of reagent solution and stirred (blank solution takes into account any minor impurities in 2,4-dinitrophenylhydrazone of formaldehyde in the reagent that formed during storage (formaldehyde exposure of the atmosphere). For sample measurement, 450 µl of deionized water was added to 50 µl of the test sample and 0.5 ml of reagent solution, and the mixture was stirred. The derivatization was performed at room temperature (22–24°C) for 20 min, after which the solution was injected into the chromatograph. For quantitative analysis, the actual calibration dependence on formaldehyde was determined using a series of solutions prepared from an analytical standard 2.4-dinitrophenylhydrazone of formaldehyde. The equation for gradient dependence was S (peak area, arbitrary units)  = 14,36×c (formaldehyde concentration in mg/l). R2 = 1. The calculation took into account the result of the blank experiment, and the formula for calculating the content of the samples was C (mg/L) = (Sx−Sblank)/14,36×20, where 14.36 is the calibration coefficient and 20 is the dilution factor.

### qRT-PCR analysis of transcript concentrations

Verification of microarray data with qRT-PCR was carried out after 4-MP intraperitoneal injection and methanol inhalation using a flow system. Mice were placed in a five-liter plastic container and blow down with air (150 liters/hour) from the evaporator, 250 ml flask with cotton wool soaked with 200 µl of methanol or water (control). One hour later, brain samples were collected after mice decapitation. RNA concentrations were determined using a Nanodrop ND-1000 spectrophotometer (Isogen Life Sciences). All RNA samples had a 260∶280 absorbance ratio between 1.9 and 2.1. cDNA was obtained by annealing 2 µg of denatured total RNA with 0.1 µg of random hexamers and 0.1 µg of Oligo-dT. The mixture was then incubated with 200 units of Superscript II reverse transcriptase (Invitrogen, USA) for 50 min at 43°C. The qRT-PCR was performed using the iCycler iQ real-time PCR detection system (Bio-Rad, Hercules, CA, USA). For the detection of target genes, the Eva Green master mix (Syntol, Russia) was used according to the manufacturer's instructions. The thermal profile for EVA Green qRT-PCR included an initial heat-denaturing step at 95°C for 3 min and 45 cycles at 95°C for 15 s, an annealing step (Table S4) for 30 sec and 72°C for 30 sec coupled with fluorescence measurements. Following amplification, the melting curves of the PCR products were monitored from 55-95°C to determine the specificity of amplification. Each sample was run in triplicate, and a non-template control was added to each run.

## Supporting Information

Figure S1 **Predicted functional interactions of the ADH/AlDH gene clusters and genes involved in cluster regulation as displayed by the STRING 9.0 database.** The predicted functional interaction networks are shown in a “confidence view”, in which the stronger associations are represented by thicker lines. Three functional protein groups are marked.
(TIF)

Table S1 **Methanol content in blood of mice after intraperitoneal methanol administration.**
(DOC)

Table S2 **The list of up-regulated genes in intersection of the Venn diagram circles presented in** **Figure 2** **.**
(DOC)

Table S3 **The list of down-regulated genes in intersection of the Venn diagram circles presented in** **Figure 2** **.**
(DOC)

Table S4 **Oligonucleotides used for qPCR.**
(DOC)
