# Supplementary figures and images for: Endogenous Methanol Regulates Mammalian Gene Activity
Source: PLoS One. 2014 Feb 27;9(2):e90239. doi: 10.1371/journal.pone.0090239 (PMC3937363; doi:10.1371/journal.pone.0090239)

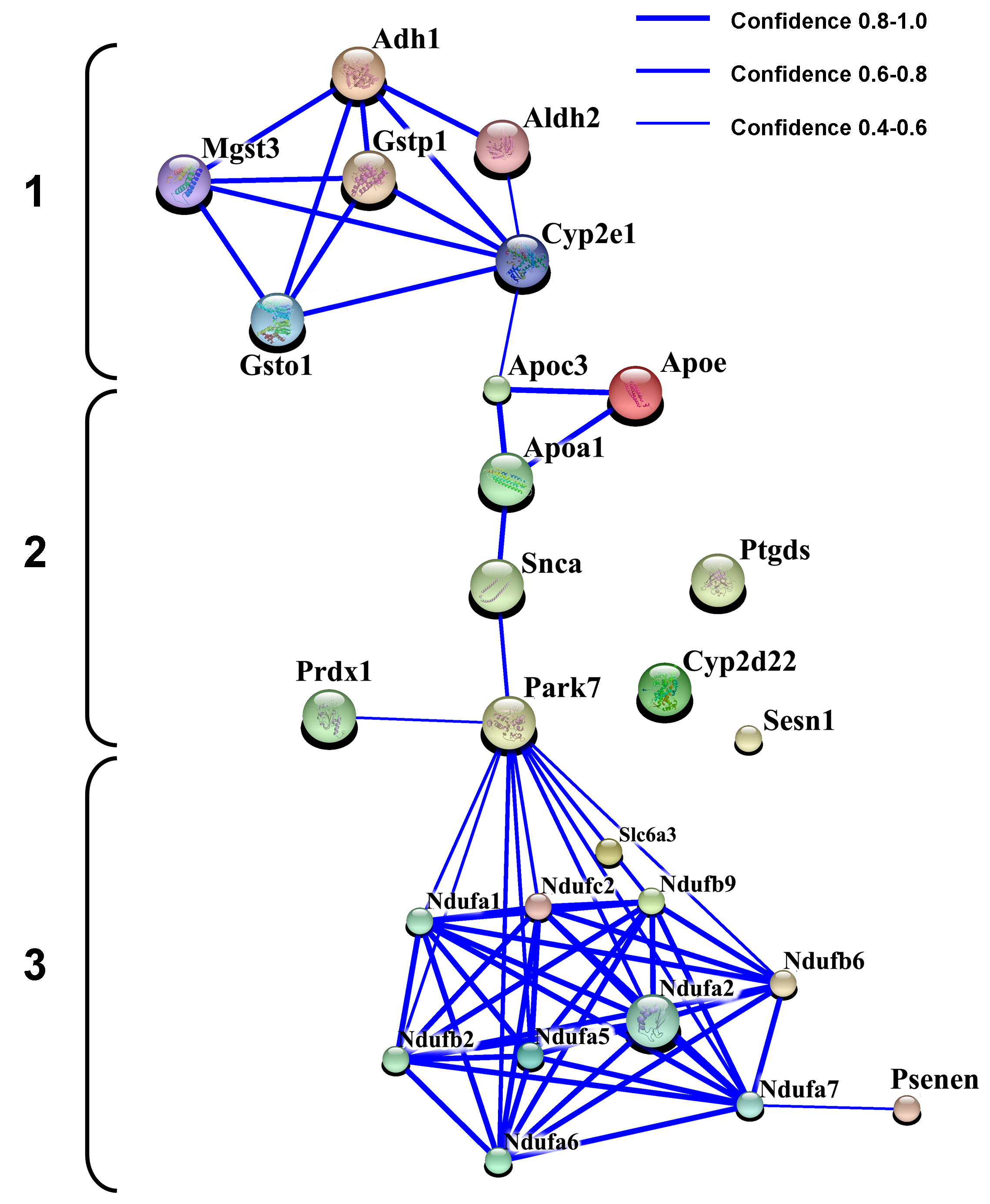

Supplement: Figure S1 — Predicted functional interactions of the ADH/AlDH gene clusters and genes involved in cluster regulation as displayed by the STRING 9.0 database. The predicted functional interaction networks are shown in a “confidence view”, in which the stronger associations are represented by thicker lines. Three functional protein groups are marked. (TIF) [file pone.0090239.s001.tif]
