# Supplementary material for: Endogenous Methanol Regulates Mammalian Gene Activity
Source: PLoS One. 2014 Feb 27;9(2):e90239. doi: 10.1371/journal.pone.0090239 (PMC3937363; doi:10.1371/journal.pone.0090239)
Supplement: Table S1 — Methanol content in blood of mice after intraperitoneal methanol administration. (DOC) [file pone.0090239.s002.doc]

Table S1. Methanol content in blood of mice after intraperitoneal methanol administration. The data with standard error bars and *P*-values (Student’s *t*-test) are indicated.

| Time after methanol administration (minutes) | Methanol concentration (µM) | *P* value (*t*-test)* |
| --- | --- | --- |
| 0 | 20.9± 1.9 | - |
| 60 | 322.1± 33.9 | <0.0001 |
| 120 | 128.1± 14.6 | <0.0001 |
| 180 | 121.3± 10.2 | <0.0001 |

* The unpaired two-tailed Student’s *t*-test shows the statistical significance of the differences between the “0” control and 60, 120 and 180 min after methanol (0.12 g/kg) administration.
