# Supplementary material for: Endogenous Methanol Regulates Mammalian Gene Activity
Source: PLoS One. 2014 Feb 27;9(2):e90239. doi: 10.1371/journal.pone.0090239 (PMC3937363; doi:10.1371/journal.pone.0090239)
Supplement: Table S2 — The list of up-regulated genes in intersection of the Venn diagram circles presented in Figure 2 . (DOC) [file pone.0090239.s003.doc]

**Table S2.** The list of up-regulated genes in intersection of the Venn diagram circles on Fig.2.

| Gene symbol | Accession number | Fold change  vs. control | *q*-value |
| --- | --- | --- | --- |
| RPL18 | NM_009077.2 | 2.305 | 0 |
| SNHG11 | NM_175692.3 | 2.250 | 0 |
| NTRK2 | NM_001025074.1 | 2.244 | 0 |
| PDHA1 | NM_008810.2 | 2.222 | 0 |
| LRRC57 | NM_025657.2 | 2.092 | 0 |
| FKBP2 | NM_008020.2 | 2.081 | 0 |
| KIF3A | NM_008443.3 | 2.075 | 0 |
| MID1IP1 | NM_026524.2 | 2.034 | 0 |
| PDRG1 | NM_178939.2 | 1.945 | 0 |
| EIF1B | NM_026892.1 | 1.922 | 0 |
| MAGED1 | NM_019791.2 | 1.921 | 0 |
| SNHG11 | NM_175692.3 | 1.918 | 0 |
| NME2 | NM_008705.4 | 1.910 | 0 |
| PDIA3 | NM_007952.2 | 1.896 | 0 |
| ARC | NM_018790.2 | 1.889 | 0 |
| THUMPD1 | NM_145585.1 | 1.885 | 0 |
| ZFP180 | NM_172483.1 | 1.852 | 0 |
| 2900060B14RIK |  | 1.826 | 0 |
| PRPF19 | NM_134129.2 | 1.794 | 0 |
| GSTP1 | NM_013541.1 | 1.782 | 0 |
| LOC100044468 | XM_001472489.1 | 1.774 | 0 |
| PPM1K | NM_175523.4 | 1.767 | 0 |
| EG666609 | XM_984926.2 | 1.754 | 0 |
| BOLA2 | NM_175103.2 | 1.728 | 0.00004 |
| SH3RF1 | NM_021506.2 | 1.725 | 0 |
| B230387C07RIK | AK046455 | 1.712 | 0 |
| EDF1 | NM_021519.1 | 1.697 | 0 |
| MGST3 | NM_025569.1 | 1.694 | 0 |
| CCDC56 | NM_026618.1 | 1.693 | 0 |
| PSENEN | NM_025498.2 | 1.688 | 0 |
| HMGN1 | NM_008251.3 | 1.687 | 0 |
| MKKS | NM_021527.1 | 1.687 | 0 |
| PDHB | NM_024221.3 | 1.687 | 0 |
| 1810027O10RIK | XM_109683.4 | 1.673 | 0 |
| PTGDS | NM_008963.1 | 1.670 | 0 |
| GALNT9 | NM_198306.1 | 1.667 | 0 |
| LOC381398 | XM_355356.1 | 1.662 | 0 |
| ANAPC5 | NM_021505.2 | 1.658 | 0 |
| TMEM14C | NM_025387.2 | 1.654 | 0 |
| XIST | NR_001463.2 | 1.650 | 0 |
| QDPR | NM_024236.1 | 1.647 | 0 |
| XIST | NR_001463.2 | 1.638 | 0 |
| LOC100047935 | XR_033756.1 | 1.635 | 0 |
| ACBD4 | NM_025988.2 | 1.633 | 0 |
| PPP1R13B | NM_011625.1 | 1.630 | 0 |
| FAM20B | NM_145413.4 | 1.624 | 0 |
| NDUFA6 | NM_025987.1 | 1.619 | 0 |
| LOC100044087 | XR_034194.1 | 1.617 | 0 |
| RESP18 | NM_009049.1 | 1.612 | 0 |
| LOC622655 | XR_032410.1 | 1.610 | 0 |
| A630082K20RIK | XM_145254 | 1.610 | 0 |
| NDRG2 | NM_013864.1 | 1.610 | 0.00241 |
| LOC232606 | XM_124826.2 | 1.607 | 0 |
| LOC546015 | XR_034790.1 | 1.607 | 0 |
| PCDH20 | NM_178685 | 1.599 | 0 |
| CCDC124 | NM_026964.3 | 1.595 | 0 |
| FAM171A2 | NM_199200.2 | 1.584 | 0 |
| DHRS7 | NM_025522.3 | 1.579 | 0 |
| PPP1R1A | NM_021391.3 | 1.576 | 0 |
| SPPL3 | NM_029012.2 | 1.575 | 0 |
| BAIAP2L1 | NM_025833.3 | 1.569 | 0 |
| A430005L14RIK | NM_175287.3 | 1.569 | 0 |
| SCHIP1 | NM_013928.4 | 1.568 | 0 |
| TRIM33 | NM_053170.2 | 1.567 | 0 |
| RPS12 | NM_011295.4 | 1.566 | 0 |
| PRKRIP1 | NM_025774.1 | 1.565 | 0 |
| NDUFB9 | NM_023172.3 | 1.565 | 0 |
| PRKAR1A | NM_021880.2 | 1.563 | 0 |
| C920006C10RIK | NM_133766.1 | 1.562 | 0 |
| COMMD10 | NM_178377.4 | 1.557 | 0 |
| GNG4 | NM_010317.2 | 1.557 | 0 |
| LOC268700 | XM_193742.2 | 1.555 | 0 |
| CUTA | NM_026307.2 | 1.554 | 0 |
| 2310004N11RIK | NM_028800 | 1.552 | 0 |
| SFRS9 | NM_025573.3 | 1.552 | 0 |
| SLC6A3 | NM_010020.3 | 1.551 | 0 |
| PCBP2 | NM_011042.1 | 1.550 | 0 |
| NDUFA5 | NM_026614.2 | 1.547 | 0 |
| ERDR1 | NM_133362.1 | 1.544 | 0 |
| OCIAD2 | NM_026950.3 | 1.542 | 0 |
| 2610507B11RIK | NM_001002004.1 | 1.541 | 0 |
| RABAC1 | NM_010261.1 | 1.541 | 0 |
| ACOT1 | NM_012006.2 | 1.539 | 0 |
| DGKZ | NM_138306.1 | 1.536 | 0 |
| 4933439C20RIK | NM_001004146.1 | 1.535 | 0 |
| GTL2 | NM_144513 | 1.535 | 0 |
| PLS3 | NM_145629.1 | 1.534 | 0 |
| 1110007L15RIK | NM_026269.1 | 1.532 | 0 |
| MRPS12 | NM_011885 | 1.531 | 0 |
| 1110001J03RIK | NM_025363.2 | 1.526 | 0 |
| B930007L02RIK | XM_356186.1 | 1.525 | 0 |
| KLC1 | NM_001025360.2 | 1.525 | 0 |
| 9030619K07RIK | AK033556 | 1.523 | 0 |
| 2410003K15RIK | XM_132537.3 | 1.520 | 0 |
| ERH | NM_007951.1 | 1.519 | 0 |
| SOX12 | NM_011438.2 | 1.518 | 0 |
| NRXN2 | NM_020253.2 | 1.518 | 0 |
| D8ERTD738E | NM_001007571.1 | 1.518 | 0.02527 |
| BANF1 | NM_001038231.1 | 1.517 | 0 |
| IFT81 | NM_009879.2 | 1.516 | 0 |
| MRPS21 | NM_078479.2 | 1.516 | 0.00616 |
| RAP2A | NM_029519.3 | 1.514 | 0 |
| BCKDHB | NM_199195.1 | 1.514 | 0 |
| UBE2G1 | NM_025985.4 | 1.512 | 0 |
| NAGK | NM_019542.1 | 1.510 | 0 |
| IFNGR2 | NM_008338.2 | 1.510 | 0 |
| 1110008F13RIK | NM_026124.2 | 1.510 | 0 |
| MEG3 | NR_003633.1 | 1.509 | 0 |
| KAT5 | NM_178637.1 | 1.508 | 0 |
| HNRPDL | NM_016690.2 | 1.508 | 0 |
| DUSP6 | NM_026268.1 | 1.507 | 0 |
| LRRC40 | NM_024194.4 | 1.507 | 0 |
| VCP | NM_009503.3 | 1.506 | 0 |
| MRPS11 | NM_026498.1 | 1.506 | 0 |
| NDUFB6 | NM_001033305.1 | 1.506 | 0 |
| GATAD1 | NM_026033.1 | 1.505 | 0 |
| SESN1 | NM_001013370.1 | 1.503 | 0 |
| ACSBG1 | NM_053178.1 | 1.503 | 0 |
| PFDN5 | NM_027044.3 | 1.503 | 0.02717 |
| OTX1 | NM_011023.3 | 1.500 | 0 |
| CSPG5 | NM_013884.2 | 1.500 | 0 |
| SCP2 | NM_011327.1 | 1.499 | 0 |
| RAPGEF1 | NM_001039086.1 | 1.499 | 0 |
| R3HDM1 | NM_181750.2 | 1.498 | 0 |
| RPAIN | NM_027186.1 | 1.497 | 0 |
| 5133401N09RIK | NM_198004.2 | 1.495 | 0.03404 |
| RPL34 | NM_026724.1 | 1.494 | 0.00594 |
| LOC676724 | XM_992449.1 | 1.494 | 0.04093 |
| 2310005L22RIK |  | 1.493 | 0 |
| RDM1 | NM_025654.2 | 1.492 | 0.00359 |
| GABRD | NM_008072.1 | 1.491 | 0 |
| UBE3C | NM_133907.3 | 1.490 | 0 |
| RPL36A | NM_019865.2 | 1.488 | 0 |
| 1110038G02RIK | AK004156 | 1.487 | 0 |
| TMEM68 | NM_028097.3 | 1.483 | 0 |
| KBTBD2 | NM_145958.1 | 1.482 | 0 |
| HES5 | NM_010419.2 | 1.481 | 0 |
| BRUNOL4 | NM_133195.2 | 1.481 | 0 |
| RBX1 | NM_019712.3 | 1.478 | 0 |
| RNF5 | NM_019403.3 | 1.476 | 0 |
| H2AFY | NM_012015.1 | 1.476 | 0 |
| 1110002E23RIK | AK003291 | 1.475 | 0 |
| TTC9B | NM_028417.1 | 1.475 | 0 |
| 1110019N10RIK | NM_026753.2 | 1.475 | 0 |
| EIF2B5 | NM_172265.1 | 1.474 | 0 |
| B930006L02RIK | NM_178764.2 | 1.474 | 0 |
| PHC1 | NM_007905.1 | 1.473 | 0 |
| TIMM8B | NM_013897.1 | 1.473 | 0.00561 |
| CNTN2 | NM_177129.5 | 1.472 | 0 |
| LOC623466 | XR_033501.1 | 1.472 | 0.00903 |
| KCNJ12 | NM_010603.4 | 1.471 | 0 |
| VTI1B | NM_016800.2 | 1.471 | 0 |
| B4GALT3 | NM_020579.1 | 1.471 | 0 |
| ATP5G2 | NM_026468.1 | 1.470 | 0 |
| SCL0002007.1_97 |  | 1.469 | 0 |
| LOC100044696 | XM_001473268.1 | 1.463 | 0 |
| UBE2M | NM_145578.1 | 1.462 | 0 |
| FAM134A | NM_170755.2 | 1.462 | 0 |
| PSMB5 | NM_011186.1 | 1.461 | 0 |
| 2410129H14RIK | NM_175245.2 | 1.459 | 0 |
| TRA2A | NM_198102.2 | 1.458 | 0 |
| SCL0004190.1_3 | AK007682.1 | 1.458 | 0 |
| PCYT1B | NM_177546.2 | 1.456 | 0 |
| CAMKV | NM_145621.2 | 1.454 | 0 |
| NOLA3 | NM_025403.2 | 1.454 | 0.00763 |
| YWHAQ | NM_011739.2 | 1.453 | 0 |
| ZCRB1 | NM_026025.1 | 1.452 | 0 |
| 6430548M08RIK | NM_172286 | 1.451 | 0 |
| PPP2R5C | NM_001081458.1 | 1.451 | 0 |
| YEATS4 | NM_026570.1 | 1.449 | 0 |
| SUMO3 | NM_019929.3 | 1.449 | 0 |
| DTYMK | NM_023136.1 | 1.449 | 0 |
| RHBDL1 | NM_144816.1 | 1.449 | 0 |
| DUSP14 | NM_019819.3 | 1.449 | 0 |
| DAB1 | NM_010014.2 | 1.449 | 0 |
| A130090K04RIK |  | 1.448 | 0 |
| PARK7 | NM_020569.1 | 1.447 | 0 |
| SLMO2 | NM_025531.2 | 1.447 | 0 |
| TUSC4 | NM_018879.1 | 1.447 | 0 |
| DCN | NM_007833.4 | 1.446 | 0 |
| PBRM1 | NM_001081251.1 | 1.445 | 0 |
| 4930573I19RIK | NM_001081057.1 | 1.445 | 0 |
| SNCA | NM_009221.2 | 1.445 | 0 |
| 1700021F05RIK | NM_026411.1 | 1.444 | 0 |
| RAN | NM_009391.3 | 1.442 | 0 |
| MAPK8IP2 | NM_021921 | 1.442 | 0 |
| NRBP2 | NM_144847.1 | 1.441 | 0 |
| PRAMEL4 | NM_001001319.3 | 1.440 | 0 |
| DLGAP4 | NM_001042487.1 | 1.439 | 0 |
| SFRS7 | NM_146083.1 | 1.439 | 0 |
| GSTO1 | NM_010362.2 | 1.438 | 0 |
| CYGB | NM_030206.1 | 1.438 | 0 |
| EGLN2 | NM_053208.2 | 1.438 | 0 |
| IMPDH2 | NM_011830 | 1.437 | 0 |
| SLC38A2 | NM_175121.3 | 1.436 | 0 |
| RPL36AL | NM_025589.1 | 1.436 | 0.00248 |
| MRPL3 | NM_053159.3 | 1.434 | 0 |
| PPA2 | NM_146141.1 | 1.434 | 0 |
| TEF | NM_017376.2 | 1.433 | 0 |
| GM561 | NM_001033297.1 | 1.433 | 0.0105 |
| LY6G6E | NM_027366.1 | 1.432 | 0 |
| LOC100042179 | XM_001477683.1 | 1.432 | 0 |
| USP22 | NM_001004143.2 | 1.430 | 0 |
| UBTD2 | NM_173784.3 | 1.426 | 0 |
| ABCA8A | NM_153145.3 | 1.425 | 0 |
| RNF6 | NM_028774.1 | 1.425 | 0.02262 |
| SQLE | NM_009270.3 | 1.423 | 0 |
| DEADC1 | NM_025748.3 | 1.423 | 0.00081 |
| GNL3 | NM_178846.1 | 1.420 | 0 |
| CLK1 | NM_001042634.1 | 1.420 | 0 |
| 5330431N19RIK | NM_172639.2 | 1.420 | 0.00554 |
| USP7 | NM_001003918.2 | 1.419 | 0 |
| MED30 | NM_027212.2 | 1.418 | 0 |
| 1700084C01RIK | NM_001033185.2 | 1.415 | 0.00114 |
| TOMM7 | NM_025394.2 | 1.415 | 0.04732 |
| ZBTB8OS | NM_025970.1 | 1.414 | 0 |
| CACNA2D1 | NM_009784.1 | 1.414 | 0 |
| MPV17 | NM_008622.1 | 1.413 | 0 |
| BRUNOL4 | NM_133195 | 1.412 | 0 |
| IL11RA1 | NM_010549.2 | 1.412 | 0.01919 |
| RUFY3 | NM_027530.2 | 1.411 | 0 |
| GRP | NM_175012.2 | 1.411 | 0 |
| CBR1 | NM_007620.2 | 1.411 | 0 |
| DDX6 | NM_007841.3 | 1.410 | 0 |
| IER3IP1 | NM_025409.1 | 1.409 | 0 |
| RPL9 | NM_011292.1 | 1.409 | 0 |
| ENSA | NM_001026212.1 | 1.408 | 0 |
| PDCD5 | NM_019746.2 | 1.408 | 0 |
| PSMD8 | NM_026545.2 | 1.408 | 0 |
| 4121402D02RIK | NM_028722.1 | 1.405 | 0 |
| BC003885 | NM_198609.2 | 1.405 | 0 |
| RPL31 | NM_053257.1 | 1.405 | 0 |
| FOS | NM_010234.2 | 1.405 | 0.00303 |
| TOX | NM_145711.3 | 1.405 | 0.00581 |
| AFG3L2 | NM_027130.1 | 1.404 | 0 |
| NECAP1 | NM_026267.2 | 1.404 | 0 |
| KATNAL1 | NM_153572.1 | 1.404 | 0 |
| THOP1 | NM_022653.3 | 1.403 | 0 |
| RND2 | NM_009708.1 | 1.403 | 0.0043 |
| RPL27A | NM_011975.3 | 1.403 | 0.00823 |
| LOC385905 | XR_034995.1 | 1.402 | 0 |
| NXT1 | NM_019761.4 | 1.402 | 0 |
| MPPED1 | NM_172610.1 | 1.401 | 0 |
| SLC35B2 | NM_028662.2 | 1.400 | 0 |
| H2AFV | XM_126043.3 | 1.399 | 0 |
| CTSZ | NM_022325.3 | 1.399 | 0 |
| 1190017O12RIK | NM_138743.2 | 1.399 | 0.0032 |
| ING4 | NM_133345.1 | 1.396 | 0 |
| CNO | NM_133724.3 | 1.396 | 0 |
| TFAM | NM_009360.2 | 1.395 | 0 |
| FCER1G | NM_010185.2 | 1.395 | 0 |
| YBX3 | AK029441 | 1.395 | 0 |
| RCHY1 | NM_026557.3 | 1.394 | 0 |
| PSMD10 | NM_016883.3 | 1.394 | 0 |
| FAM152B | NM_134095.2 | 1.393 | 0 |
| SCRN1 | NM_027268 | 1.393 | 0 |
| BEX2 | NM_009749.1 | 1.392 | 0 |
| RSN | NM_019765 | 1.391 | 0 |
| MRPS16 | NM_025440.2 | 1.391 | 0.00103 |
| PRDX1 | NM_011034.4 | 1.390 | 0 |
| TCF4 | NM_013685.2 | 1.389 | 0 |
| UBQLN1 | NM_026842.3 | 1.389 | 0 |
| GPR85 | NM_145066.4 | 1.389 | 0 |
| PMVK | NM_026784.2 | 1.389 | 0 |
| KLF6 | NM_011803.2 | 1.389 | 0 |
| CLK4 | NM_007714.2 | 1.388 | 0 |
| ZXDA | NR_003292.1 | 1.386 | 0 |
| MYO5B | NM_201600.2 | 1.385 | 0 |
| KRAS | NM_021284.4 | 1.385 | 0 |
| SFRS2 | NM_011358.1 | 1.384 | 0 |
| PHF13 | NM_172705.1 | 1.384 | 0 |
| SEPT6 | NM_019942.4 | 1.383 | 0 |
| GLTP | NM_019821.2 | 1.382 | 0 |
| TSR2 | NM_175146.2 | 1.380 | 0 |
| HCRT | NM_010410.2 | 1.38 | 0.00007 |
| BMPER | NM_028472.1 | 1.379 | 0 |
| ZCCHC12 | NM_028325.2 | 1.379 | 0 |
| EEF1A2 | NM_007906.2 | 1.379 | 0 |
| HDHD3 | NM_024257.1 | 1.379 | 0 |
| RPL27A | NM_011975.3 | 1.378 | 0 |
| PHCA | NM_025408.2 | 1.377 | 0 |
| CRY2 | NM_009963.3 | 1.375 | 0 |
| MSRB2 | NM_029619.2 | 1.375 | 0 |
| B4GALT6 | NM_019737.1 | 1.374 | 0 |
| EEF1B2 | NM_018796.2 | 1.374 | 0.00757 |
| RFWD2 | NM_011931.3 | 1.372 | 0 |
| NSFL1C | NM_198326.2 | 1.372 | 0 |
| EG668850 | XR_002259.2 | 1.372 | 0 |
| 2010005J08RIK | NM_178623.2 | 1.371 | 0 |
| ABR | NM_198894.1 | 1.371 | 0 |
| ATP9B | NM_015805.2 | 1.370 | 0 |
| SNRPD1 | NM_009226.2 | 1.370 | 0 |
| ZMYM2 | NM_029498.2 | 1.370 | 0 |
| LOC433546 | XM_906947.2 | 1.370 | 0 |
| CALM3 | NM_007590.3 | 1.369 | 0 |
| ATXN10 | NM_016843.3 | 1.369 | 0 |
| ROGDI | NM_133185.2 | 1.367 | 0 |
| CLK4 | NM_007714.4 | 1.367 | 0 |
| NRIP3 | NM_020610.1 | 1.366 | 0 |
| LASP1 | NM_010688.2 | 1.366 | 0 |
| SLC25A38 | NM_144793.1 | 1.366 | 0 |
| 0610007J10RIK | AK018717 | 1.366 | 0.02851 |
| C630004H02RIK | NM_175454.2 | 1.365 | 0 |
| SIAH1A | NM_009172.1 | 1.365 | 0 |
| DLGAP1 | NM_177639.5 | 1.365 | 0 |
| KRT75 | NM_133357.1 | 1.364 | 0 |
| LOC639931 | XM_916567.2 | 1.363 | 0 |
| PRKCBP1 | NM_027230.3 | 1.363 | 0 |
| OCIAD2 | NM_026950.3 | 1.362 | 0 |
| MEGF9 | NM_172694.2 | 1.362 | 0 |
| RHOT2 | NM_145999.2 | 1.362 | 0 |
| GNB5 | NM_010313.1 | 1.362 | 0 |
| MFAP3 | NM_145426.2 | 1.361 | 0 |
| GNA11 | NM_010301.3 | 1.361 | 0 |
| ELOF1 | NM_170777.3 | 1.361 | 0 |
| ARMCX2 | NM_026139.3 | 1.361 | 0 |
| RPL13A | NM_009438.3 | 1.361 | 0 |
| 2810408P10RIK | NM_198619.2 | 1.360 | 0 |
| 4933427G23RIK | XR_005167.1 | 1.359 | 0 |
| SNRPG | NM_026506.1 | 1.359 | 0 |
| 5430405G05RIK | AK077378 | 1.358 | 0 |
| UQCRH | NM_025641.3 | 1.358 | 0 |
| MYO5A | NM_010864.2 | 1.358 | 0 |
| RASA2 | NM_053268.2 | 1.357 | 0 |
| STATIP1 | NM_021448.1 | 1.357 | 0 |
| IAP | NM_010490.2 | 1.357 | 0 |
| LOC634015 | XR_003906.1 | 1.357 | 0 |
| NTNG1 | NM_030699.1 | 1.357 | 0.00521 |
| SRI | NM_025618.2 | 1.356 | 0 |
| SLC39A7 | NM_008202.2 | 1.356 | 0 |
| LOC433955 | XR_033167.1 | 1.356 | 0 |
| NME7 | NM_138314.2 | 1.355 | 0 |
| SEC61B | NM_024171.1 | 1.354 | 0.02673 |
| MTVR2 | NM_023166 | 1.353 | 0 |
| VKORC1 | NM_178600.2 | 1.353 | 0 |
| ADORA1 | NM_001008533.2 | 1.352 | 0 |
| CHURC1 | NM_206534.1 | 1.352 | 0 |
| ANKRD12 | NM_001025572.1 | 1.352 | 0 |
| PYGO2 | XM_130932.5 | 1.351 | 0 |
| EHBP1L1 | NM_053252.2 | 1.351 | 0 |
| ABHD3 | NM_134130.1 | 1.350 | 0 |
| BAP1 | NM_027088.1 | 1.350 | 0 |
| 4932408L24RIK | XM_162190.1 | 1.349 | 0 |
| LIX1 | NM_025681.2 | 1.349 | 0 |
| EIF3S5 | NM_025344 | 1.349 | 0 |
| SIRT1 | NM_019812.1 | 1.347 | 0 |
| CYP2D22 | NM_019823.3 | 1.347 | 0 |
| CIB2 | NM_019686.3 | 1.347 | 0 |
| CHIC2 | NM_028850.1 | 1.346 | 0 |
| MFN2 | NM_133201.2 | 1.346 | 0 |
| WSB1 | NM_001042565.2 | 1.346 | 0 |
| USE1 | NM_025917.3 | 1.346 | 0 |
| CHRNA4 | NM_015730.4 | 1.345 | 0 |
| RBBP7 | NM_009031.2 | 1.345 | 0 |
| CAR14 | NM_011797.1 | 1.345 | 0.01185 |
| EG626175 | XM_908326.3 | 1.345 | 0.01375 |
| RPS8 | NM_009098.2 | 1.345 | 0.02498 |
| ATP5L | NM_013795.4 | 1.344 | 0 |
| 4930470O13RIK |  | 1.343 | 0 |
| HGFAC | NM_019447.1 | 1.343 | 0 |
| AI646023 | NM_198860.1 | 1.343 | 0 |
| B020018G12RIK | NM_001039518.1 | 1.343 | 0 |
| SRI | NM_001080974.1 | 1.342 | 0 |
| TMEM11 | NM_173453.2 | 1.342 | 0 |
| C1QBP | NM_007573.2 | 1.342 | 0 |
| ABCF2 | NM_013853.1 | 1.342 | 0 |
| CRYZL1 | NM_133679.1 | 1.341 | 0 |
| PCNXL2 | NM_175561.3 | 1.341 | 0 |
| PSMB1 | NM_011185.3 | 1.341 | 0 |
| KHDRBS3 | NM_010158.2 | 1.340 | 0 |
| GPD1 | NM_010271.2 | 1.340 | 0 |
| GNAT1 | NM_008140.2 | 1.339 | 0 |
| HMG20A | NM_025812.2 | 1.339 | 0 |
| IARS | NM_172015.1 | 1.337 | 0 |
| RPS5 | NM_009095.1 | 1.337 | 0.0087 |
| SLC23A3 | NM_194333.3 | 1.336 | 0 |
| MEG3 | NR_003633.1 | 1.336 | 0 |
| RPL23 | NM_022891.2 | 1.336 | 0.01352 |
| RAB11FIP4 | NM_175543 | 1.335 | 0 |
| ARL3 | NM_019718.2 | 1.335 | 0 |
| DPM2 | NM_010073.2 | 1.334 | 0 |
| TMEM38B | NM_028053.1 | 1.333 | 0 |
| SCGN | NM_145399.1 | 1.333 | 0 |
| 1300001I01RIK | NM_001081158.2 | 1.333 | 0 |
| MAGOH | NM_010760.2 | 1.333 | 0 |
| GPR34 | NM_011823.4 | 1.332 | 0 |
| CLK1 | NM_009905.1 | 1.332 | 0 |
| 1200015N20RIK | NM_024244 | 1.332 | 0 |
| CFDP1 | NM_011801.1 | 1.332 | 0 |
| FSCN1 | NM_007984.2 | 1.332 | 0 |
| RAB28 | NM_027295.1 | 1.331 | 0 |
| AAMP | NM_146110.1 | 1.331 | 0 |
| RPS5 | NM_009095 | 1.331 | 0.0062 |
| GUK1 | NM_008193 | 1.331 | 0.01115 |
| KLHL21 | NM_001033352.3 | 1.330 | 0 |
| CCDC80 | NM_026439.2 | 1.330 | 0 |
| DPH2 | NM_026344.3 | 1.330 | 0 |
| ATF4 | NM_009716.2 | 1.330 | 0.00192 |
| KIF3A | NM_008443.3 | 1.329 | 0 |
| ZFP523 | NM_172617.1 | 1.329 | 0 |
| TSEN15 | NM_025677.2 | 1.329 | 0 |
| ENSMUSG00000054212 | NM_145433.1 | 1.329 | 0 |
| PSMC3 | NM_008948.1 | 1.329 | 0.02548 |
| 4930455C21RIK | NM_024273.1 | 1.328 | 0 |
| RPL39 | NM_026055.1 | 1.328 | 0 |
| GFOD1 | NM_001033399.1 | 1.328 | 0 |
| A030007L17RIK | NM_026637.3 | 1.327 | 0 |
| 0610012G03RIK | NM_025320 | 1.327 | 0 |
| PPFIA3 | NM_029741.1 | 1.327 | 0 |
| 2010007H12RIK | NM_027242.3 | 1.327 | 0 |
| TPPP | NM_182839.1 | 1.326 | 0 |
| ARMC8 | NM_028768.2 | 1.326 | 0 |
| HYI | NM_026601.1 | 1.326 | 0.01904 |
| GATS | NM_030719.3 | 1.325 | 0 |
| NDUFC2 | NM_024220.1 | 1.325 | 0 |
| WIPF2 | NM_197940.1 | 1.325 | 0 |
| JTV1 | NM_146165.1 | 1.325 | 0 |
| CORO1B | NM_011778.1 | 1.324 | 0 |
| GLT8D1 | NM_029626.1 | 1.324 | 0 |
| STK25 | NM_021537.2 | 1.324 | 0 |
| NUDT9 | NM_028794.3 | 1.323 | 0 |
| CSNK1G2 | NM_134002.1 | 1.323 | 0 |
| TIA1 | AK009502 | 1.323 | 0 |
| ORMDL3 | NM_025661.3 | 1.322 | 0 |
| PREB | NM_016703.1 | 1.322 | 0 |
| AI256775 | NM_178916 | 1.321 | 0 |
| H2-D1 | NM_010380.2 | 1.321 | 0 |
| NUBP1 | NM_011955.1 | 1.321 | 0 |
| SOD1 | NM_011434.1 | 1.321 | 0.00139 |
| 2900097C17RIK | XM_001480665.1 | 1.321 | 0.02151 |
| 1110021J02RIK |  | 1.321 | 0.02744 |
| RPP38 | XM_130010.3 | 1.320 | 0 |
| NXPH1 | NM_008751.2 | 1.320 | 0 |
| B3GAT3 | NM_024256.2 | 1.320 | 0 |
| TPRKB | NM_176842.2 | 1.320 | 0.00081 |
| 100041294 | NM_011509.1 | 1.320 | 0.01103 |
| LOC667251 | XR_032081.1 | 1.318 | 0 |
| LRRC7 | XM_993731.1 | 1.318 | 0.00092 |
| COMT | XM_147265.1 | 1.317 | 0 |
| NNAT | NM_180960.2 | 1.316 | 0 |
| 1500011K16RIK | XR_035441.1 | 1.316 | 0.00286 |
| 2600009E05RIK | NM_029832.1 | 1.315 | 0 |
| PIK3CA | NM_008839.1 | 1.314 | 0 |
| 1700020C11RIK | NM_026443.4 | 1.314 | 0 |
| FKTN | NM_139309.4 | 1.314 | 0 |
| DHX30 | NM_133347.1 | 1.314 | 0 |
| ZC3H14 | NM_029334.1 | 1.314 | 0 |
| EG216185 | XM_911906.3 | 1.314 | 0.00828 |
| RPS24 | NM_207634.1 | 1.313 | 0 |
| CCNG1 | NM_009831.2 | 1.313 | 0 |
| SLC17A7 | NM_182993.1 | 1.312 | 0 |
| PER1 | NM_011065.2 | 1.312 | 0 |
| PRKG2 | NM_008926.3 | 1.312 | 0 |
| 1110008P14RIK | NM_198001.1 | 1.312 | 0 |
| THOC1 | NM_153552.2 | 1.311 | 0 |
| 2610030H06RIK | XM_110750 | 1.311 | 0 |
| GLRX5 | NM_028419.2 | 1.311 | 0 |
| IGSF21 | NM_198610.1 | 1.310 | 0 |
| OLFR679 | NM_147044.1 | 1.310 | 0 |
| CDH22 | NM_174988.2 | 1.310 | 0 |
| RPL12 | NM_009076.1 | 1.310 | 0.02842 |
| TFB1M | NM_146074.1 | 1.309 | 0 |
| MRPS10 | NM_183086 | 1.309 | 0 |
| RASGRF1 | NM_011245.1 | 1.309 | 0.00673 |
| TMEM111 | NM_175101.2 | 1.308 | 0 |
| 1110008J03RIK | NM_029096.2 | 1.308 | 0 |
| PIGQ | NM_011822.3 | 1.308 | 0 |
| SCGN | NM_145399.1 | 1.308 | 0 |
| TMCC2 | NM_178874.2 | 1.308 | 0.01508 |
| ELAVL2 | NM_207686.1 | 1.307 | 0 |
| GOLGA7B | NM_027694.1 | 1.307 | 0 |
| TMEM14C | NM_025387.2 | 1.307 | 0 |
| B230333C21RIK | XM_111900.3 | 1.306 | 0 |
| SREBF2 | AK032847 | 1.306 | 0 |
| B430305P08RIK | XM_148123.1 | 1.306 | 0 |
| HABP4 | NM_019986.1 | 1.306 | 0 |
| H47 | NM_024439.3 | 1.305 | 0 |
| 1810043G02RIK | NM_026431.2 | 1.305 | 0 |
| SMAD3 | NM_016769.2 | 1.305 | 0.04584 |
| RAB24 | NM_009000.3 | 1.304 | 0 |
| HRSP12 | NM_008287.2 | 1.304 | 0 |
| NUDT19 | NM_033080.2 | 1.304 | 0.02504 |
| TSPAN6 | NM_019656.3 | 1.303 | 0 |
| SCARA3 | NM_172604.3 | 1.303 | 0 |
| FOSB | NM_008036.2 | 1.303 | 0 |
| PSMB7 | NM_011187.1 | 1.303 | 0 |
| MAP1LC3A | NM_025735.1 | 1.303 | 0 |
| FBXL18 | NM_001033312.3 | 1.303 | 0 |
| GPX3 | NM_008161.2 | 1.303 | 0.00463 |
| FMN2 | NM_019445.1 | 1.303 | 0.03007 |
| SKP2 | NM_145468.1 | 1.302 | 0 |
| AP1S1 | NM_007457.2 | 1.302 | 0 |
| FSTL1 | NM_008047.4 | 1.302 | 0 |
| A830059I20RIK | NM_021427.1 | 1.301 | 0 |
| IMPDH1 | NM_011829.2 | 1.301 | 0 |
| LOC280205 | XM_207178.1 | 1.301 | 0.02539 |
| DLX2 | NM_010054.1 | 1.300 | 0 |
