# Supplementary material for: Endogenous Methanol Regulates Mammalian Gene Activity
Source: PLoS One. 2014 Feb 27;9(2):e90239. doi: 10.1371/journal.pone.0090239 (PMC3937363; doi:10.1371/journal.pone.0090239)
Supplement: Table S3 — The list of down-regulated genes in intersection of the Venn diagram circles presented in Figure 2 . (DOC) [file pone.0090239.s004.doc]

**Table S3**. The list of down-regulated genes in intersection of the Venn diagram circles on Fig. 2.

| Gene symbol | Accession number | Fold change  vs. control | *q*-value |
| --- | --- | --- | --- |
| MT-ATP6 |  | -1.300 | 0 |
| TUBA3B | NM_009449.3 | -1.300 | 0 |
| EIF4A1 | NM_144958.3 | -1.300 | 0 |
| LOC630936 | XR_001819.2 | -1.301 | 0 |
| PMPCB | NM_028431.2 | -1.302 | 0 |
| TTC15 | NM_178811.3 | -1.302 | 0 |
| TESK1 | NM_011571.2 | -1.302 | 0 |
| ST8SIA5 | NM_153124.1 | -1.302 | 0 |
| OLFR620 | NM_146812.1 | -1.302 | 0 |
| SH3GLB1 | NM_019464.2 | -1.303 | 0 |
| TRPC4AP | NM_019828.1 | -1.303 | 0 |
| PIK3R3 | NM_181585.5 | -1.303 | 0 |
| OLFR1107 | NM_146844.1 | -1.304 | 0 |
| BCLAF1 | NM_001025393.1 | -1.304 | 0 |
| LHX1 | NM_008498 | -1.304 | 0.01256 |
| DCTN2 | NM_027151.1 | -1.305 | 0 |
| 6720458D17RIK |  | -1.306 | 0 |
| CD74 | NM_001042605.1 | -1.306 | 0 |
| 2400003C14RIK | NM_028018.1 | -1.307 | 0 |
| GLUL | NM_008131.3 | -1.307 | 0 |
| AKAP9 | NM_194462.1 | -1.307 | 0 |
| DAG1 | NM_010017.3 | -1.308 | 0 |
| ERMP1 | NM_001081213.1 | -1.308 | 0 |
| COL18A1 | NM_009929.2 | -1.308 | 0 |
| KCNAB1 | NM_010597.3 | -1.308 | 0 |
| CXCL9 | NM_008599.3 | -1.309 | 0 |
| ZDHHC13 | NM_028031.3 | -1.309 | 0 |
| PFKP | NM_019703.3 | -1.310 | 0.0046 |
| TXNDC5 | NM_145367.3 | -1.311 | 0 |
| SLC1A3 | NM_148938.2 | -1.312 | 0 |
| NNAT | NM_010923.2 | -1.313 | 0 |
| IRF1 | NM_008390.1 | -1.314 | 0 |
| PCDH10 | NM_011043.3 | -1.315 | 0 |
| CSF1R | NM_001037859.2 | -1.315 | 0 |
| D1BWG0212E | NM_028043.2 | -1.315 | 0 |
| 2610024H22RIK |  | -1.315 | 0.00362 |
| DHX9 | NM_007842.2 | -1.316 | 0 |
| RP9 | NM_018739.1 | -1.316 | 0 |
| ITGB4 | NM_133663.2 | -1.316 | 0 |
| 4833426J09RIK | NM_001024606.1 | -1.317 | 0 |
| PHACTR4 | NM_175306.3 | -1.317 | 0 |
| CUL3 | NM_016716 | -1.317 | 0 |
| PNPLA2 | NM_025802.2 | -1.318 | 0 |
| MTUS1 | NM_001005864.2 | -1.318 | 0 |
| RGS17 | NM_019958.3 | -1.318 | 0 |
| CYFIP2 | NM_133769.2 | -1.319 | 0 |
| LRRC49 | NM_145616.2 | -1.319 | 0 |
| PDE4B | NM_019840.2 | -1.320 | 0 |
| GAS7 | NM_008088.1 | -1.320 | 0 |
| SPATA13 | XM_147847.4 | -1.320 | 0 |
| 6330527O06RIK | NM_029530.2 | -1.321 | 0 |
| TNS3 | NM_001083587.1 | -1.321 | 0 |
| MCM5 | NM_008566.2 | -1.322 | 0 |
| MTMR2 | NM_023858.1 | -1.322 | 0 |
| MCM6 | NM_008567.1 | -1.324 | 0 |
| KIF3B | NM_008444 | -1.324 | 0 |
| CAMK4 | NM_009793 | -1.325 | 0 |
| EG433365 | NM_001008426.1 | -1.326 | 0 |
| CDC5L | NM_152810.1 | -1.326 | 0 |
| SLC24A5 | NM_175034.3 | -1.327 | 0 |
| TBCD | NM_029878.1 | -1.327 | 0 |
| LRCH1 | NM_001033439.2 | -1.327 | 0 |
| SPSB1 | NM_029035.2 | -1.327 | 0 |
| PDCD4 | NM_011050.3 | -1.327 | 0 |
| DNM1 | NM_010065.2 | -1.327 | 0 |
| PEX10 | NM_001042407.1 | -1.328 | 0 |
| NCAN | NM_007789.2 | -1.328 | 0 |
| ERRFI1 | NM_133753.1 | -1.330 | 0 |
| CIRBP | NM_007705.2 | -1.330 | 0 |
| SEMA6B | XM_001004114.1 | -1.330 | 0 |
| CPM | XM_994613.1 | -1.331 | 0 |
| 3110001P07RIK | AK013971 | -1.331 | 0 |
| CEP120 | NM_178686.3 | -1.332 | 0 |
| SQSTM1 | NM_011018.2 | -1.332 | 0 |
| TSPYL3 | NM_198617.1 | -1.332 | 0 |
| TXNDC13 | NM_029148.1 | -1.332 | 0 |
| 1600021P15RIK | NM_177718.3 | -1.333 | 0 |
| H13 | NM_010376.3 | -1.333 | 0 |
| ARRDC3 | NM_178917.2 | -1.334 | 0 |
| TIMP3 | NM_011595.2 | -1.335 | 0 |
| CDKN1A | NM_007669.2 | -1.335 | 0 |
| ANXA6 | NM_013472.2 | -1.336 | 0 |
| PDXDC1 | NM_001039533.1 | -1.336 | 0 |
| GNG12 | AK005561 | -1.337 | 0 |
| LOC100047353 | XM_001477963.1 | -1.337 | 0.00769 |
| ZCCHC3 | NM_175126.3 | -1.338 | 0 |
| GPHN | NM_172952.2 | -1.338 | 0 |
| EIF2AK2 | NM_011163.3 | -1.339 | 0 |
| ALAS1 | NM_020559.1 | -1.339 | 0 |
| VPS25 | NM_026776.3 | -1.339 | 0 |
| SPARCL1 | NM_010097.2 | -1.340 | 0 |
| CHST2 | NM_018763.1 | -1.340 | 0 |
| BC003331 | NM_145511.1 | -1.340 | 0 |
| SRXN1 | NM_029688.4 | -1.342 | 0 |
| ANKRD13A | NM_026718.2 | -1.343 | 0 |
| USP2 | NM_198091.2 | -1.344 | 0 |
| GRIT | NM_177379.2 | -1.345 | 0 |
| GADD45G | NM_011817.1 | -1.345 | 0 |
| ITM2B | NM_008410.1 | -1.346 | 0 |
| EG665685 | XR_001769.2 | -1.346 | 0 |
| LOC673501 | XM_001005723.1 | -1.347 | 0.01793 |
| HPS5 | NM_001005248.1 | -1.348 | 0 |
| H2-AB1 | NM_207105.1 | -1.348 | 0 |
| LMO7 | XM_895783.2 | -1.349 | 0 |
| UNC84B | NM_194342.1 | -1.352 | 0 |
| RIMS2 | NM_053271.1 | -1.353 | 0 |
| KLF9 | NM_010638.4 | -1.353 | 0 |
| IFI47 | NM_008330.1 | -1.354 | 0 |
| NOSTRIN | NM_181547.2 | -1.355 | 0 |
| 0910001A06RIK | NM_144846.4 | -1.355 | 0 |
| CLIC6 | NM_172469.3 | -1.356 | 0 |
| LOC677317 | XM_001004685.1 | -1.356 | 0 |
| UCK1 | NM_011675.1 | -1.356 | 0 |
| SLC10A4 | NM_173403.2 | -1.358 | 0 |
| SRXN1 | NM_029688.2 | -1.359 | 0 |
| ANGPT2 | NM_007426.3 | -1.359 | 0 |
| RNF103 | NM_009543.1 | -1.360 | 0 |
| UGT8A | NM_011674.4 | -1.361 | 0 |
| LOC100047619 | XR_033736.1 | -1.362 | 0 |
| AKAP9 | NM_194462.2 | -1.362 | 0 |
| PTPRA | NM_008980.1 | -1.363 | 0 |
| CUTA | NM_026307.2 | -1.363 | 0 |
| ROBO4 | NM_028783.2 | -1.365 | 0 |
| MT2 | NM_008630.2 | -1.367 | 0 |
| HSPB6 | NM_001012401.1 | -1.367 | 0 |
| MTDNA_ND4L |  | -1.371 | 0 |
| WASF2 | NM_153423 | -1.372 | 0 |
| H3F3B | NM_008211.3 | -1.373 | 0 |
| RARB | NM_011243.1 | -1.373 | 0.00735 |
| FFAR3 | NM_001033316.2 | -1.374 | 0 |
| KCNA5 | NM_145983.1 | -1.374 | 0 |
| GSPT1 | NM_146066.1 | -1.375 | 0 |
| D1BWG0212E | NM_028043.2 | -1.375 | 0 |
| SF3B2 | NM_030109.1 | -1.376 | 0 |
| TPI1 | NM_009415.1 | -1.376 | 0 |
| MTA3 | NM_054082.1 | -1.376 | 0 |
| FAM134B | NM_025459.2 | -1.376 | 0 |
| MPP3 | NM_007863.1 | -1.377 | 0 |
| CTSA | NM_001038492.1 | -1.377 | 0 |
| GNAQ | NM_008139.5 | -1.377 | 0.02673 |
| CLCN3 | NM_007711.2 | -1.378 | 0.00406 |
| HIST1H1C | NM_015786 | -1.380 | 0 |
| ALG2 | NM_019998.2 | -1.381 | 0 |
| LOC100046746 | XM_001476743.1 | -1.381 | 0 |
| LOC676136 | XM_987671.1 | -1.382 | 0 |
| AKT1S1 | NM_026270.3 | -1.383 | 0 |
| TBC1D8 | NM_018775.3 | -1.383 | 0 |
| EXTL2 | NM_021388.3 | -1.384 | 0 |
| BC024868 | NM_199149.1 | -1.385 | 0 |
| IFIT3 | NM_010501.1 | -1.385 | 0 |
| PCOLN3 | NM_145606.1 | -1.386 | 0 |
| ANXA3 | NM_013470.1 | -1.386 | 0 |
| DNAJC16 | NM_172338.2 | -1.388 | 0 |
| LOC100047788 | XR_033948.1 | -1.388 | 0 |
| RASD2 | XM_204287.8 | -1.389 | 0.00718 |
| LOC100046254 | XM_001475816.1 | -1.390 | 0 |
| ADIPOR2 | NM_197985.2 | -1.392 | 0 |
| PRICKLE1 | NM_001033217.3 | -1.393 | 0 |
| APLP2 | NM_009691.2 | -1.393 | 0.03026 |
| F2R | NM_010169.3 | -1.398 | 0 |
| CLDN11 | NM_008770.2 | -1.400 | 0 |
| TAPBP | NM_001025313.1 | -1.400 | 0 |
| LOC272693 | XM_195425.1 | -1.402 | 0 |
| SCN4B | NM_001013390.2 | -1.411 | 0 |
| IGSF8 | NM_080419.1 | -1.412 | 0 |
| AB023957 | NM_133237.1 | -1.412 | 0 |
| EXOC7 | NM_016857.1 | -1.412 | 0 |
| VAPB | NM_019806.5 | -1.413 | 0.03923 |
| LOC667370 | XM_001480084.1 | -1.414 | 0 |
| PENK1 | NM_001002927.2 | -1.414 | 0.01486 |
| DDIT4 | NM_029083.1 | -1.415 | 0 |
| KLF7 | AK086122 | -1.415 | 0 |
| APOE | NM_009696.2 | -1.419 | 0 |
| SERPINA3H | NM_001034870.2 | -1.420 | 0 |
| PDXDC1 | NM_001039533.1 | -1.420 | 0 |
| PKP4 | NM_175464.2 | -1.422 | 0 |
| TFRC | NM_011638.3 | -1.423 | 0 |
| PIP4K2A | NM_008845.4 | -1.425 | 0 |
| COX10 | NM_178379.2 | -1.425 | 0 |
| CEND1 | NM_021316.2 | -1.426 | 0 |
| GALNTL2 | NM_030166.1 | -1.427 | 0 |
| PDYN | NM_018863.2 | -1.428 | 0.04204 |
| RBM14 | NM_019869.2 | -1.430 | 0 |
| ENDOD1 | NM_028013.2 | -1.433 | 0 |
| SLC6A11 | NM_172890.3 | -1.433 | 0 |
| STMN4 | NM_019675.2 | -1.433 | 0.01814 |
| NCAM1 | NM_010875.3 | -1.443 | 0 |
| NRGN | NM_022029.2 | -1.446 | 0 |
| MAL | NM_010762.4 | -1.447 | 0 |
| RBBP9 | NM_015754.2 | -1.447 | 0 |
| TRPC3 | NM_019510.1 | -1.448 | 0 |
| LOC385068 | XM_358030.1 | -1.448 | 0 |
| TWISTNB | NM_172253.2 | -1.449 | 0 |
| ACTN2 | NM_033268.2 | -1.449 | 0 |
| LOC100046207 | XM_001475801.1 | -1.451 | 0 |
| FA2H | NM_178086.2 | -1.452 | 0 |
| ENPP2 | NM_015744.1 | -1.453 | 0 |
| MAT2A | NM_145569.4 | -1.454 | 0 |
| GPR17 | NM_001025381.1 | -1.456 | 0 |
| DDX21 | NM_019553.2 | -1.457 | 0 |
| H2-D1 | NM_010380.3 | -1.457 | 0 |
| FGFR1OP2 | NM_026218.2 | -1.459 | 0 |
| NKD1 | NM_027280.2 | -1.463 | 0 |
| TXNIP | NM_023719.1 | -1.463 | 0 |
| SLC4A4 | NM_018760.1 | -1.464 | 0 |
| VPS53 | NM_026664.2 | -1.464 | 0 |
| CLCN3 | NM_007711.2 | -1.466 | 0 |
| BCAS1 | NM_029815.1 | -1.466 | 0 |
| PDE1B | NM_008800.1 | -1.466 | 0 |
| C630013B14RIK | AK049936 | -1.471 | 0 |
| H2-T23 | NM_010398.1 | -1.473 | 0 |
| REEP3 | NM_178606.4 | -1.481 | 0 |
| C430004E15RIK | NM_175286.3 | -1.481 | 0 |
| ENPP2 | NM_015744 | -1.484 | 0 |
| ACTB | NM_007393.1 | -1.490 | 0.01486 |
| ELTD1 | NM_133222.2 | -1.491 | 0 |
| ATP2A3 | NM_016745.2 | -1.492 | 0 |
| IIGP2 | NM_019440.2 | -1.496 | 0 |
| LOC331139 | XM_284587.2 | -1.500 | 0 |
| PHACTR1 | NM_001005740.1 | -1.515 | 0 |
| LOC669053 | XM_972773.1 | -1.516 | 0 |
| GRID2 | NM_008167 | -1.517 | 0 |
| H1F0 | NM_008197.3 | -1.519 | 0 |
| LOC674135 | XR_004604.2 | -1.519 | 0 |
| TPI1 | NM_009415.1 | -1.526 | 0 |
| GNG7 | NM_010319.3 | -1.530 | 0 |
| USP29 | NM_021323.2 | -1.533 | 0 |
| PDE1B | NM_008800 | -1.534 | 0 |
| S3-12 | NM_020568.2 | -1.537 | 0 |
| GDF10 | NM_145741.2 | -1.545 | 0 |
| LOC641240 | XM_918601.3 | -1.559 | 0 |
| RGS9 | NM_011268.2 | -1.560 | 0 |
| PSMB9 | NM_013585.2 | -1.561 | 0 |
| CNTFR | NM_016673.1 | -1.564 | 0 |
| GCNT2 | NM_133219.1 | -1.565 | 0 |
| DCLK3 | NM_172928.3 | -1.566 | 0 |
| FLT1 | NM_010228.3 | -1.567 | 0 |
| ATP2A3 | NM_016745.2 | -1.571 | 0 |
| REM2 | NM_080726.3 | -1.573 | 0 |
| ESAM | NM_027102.2 | -1.576 | 0 |
| B2M | NM_009735.2 | -1.586 | 0 |
| а | NM_007669.3 | -1.601 | 0 |
| EG667977 | NM_001081032.1 | -1.603 | 0 |
| HSPA1B | NM_010478.2 | -1.606 | 0 |
| TWISTNB | NM_172253.2 | -1.636 | 0 |
| IGFBP7 | NM_008048.2 | -1.654 | 0 |
| MOG | NM_010814.2 | -1.659 | 0 |
| MAL | NM_010762.4 | -1.663 | 0 |
| POP4 | NM_025390.4 | -1.663 | 0 |
| LY6A | NM_010738.2 | -1.665 | 0 |
| STK32A | NM_178749 | -1.682 | 0 |
| MOBP | NM_001039365.1 | -1.684 | 0 |
| OASL2 | NM_011854.1 | -1.695 | 0 |
| GPR88 | NM_022427.1 | -1.708 | 0 |
| ACTA2 | NM_007392.2 | -1.711 | 0 |
| MOBP | AK013799 | -1.738 | 0 |
| IGK-V33 | XM_144817.1 | -1.751 | 0 |
| H2-T23 | NM_010398.1 | -1.757 | 0 |
| LOC100043918 | XM_001481237.1 | -1.765 | 0 |
| IRGM1 | NM_008326.1 | -1.767 | 0 |
| LOC243439 | XM_992249.1 | -1.792 | 0 |
| PPP1R1B | NM_144828.1 | -1.797 | 0 |
| LOC229810 | XM_124173.2 | -1.815 | 0 |
| LOC633016 | XM_921371.2 | -1.823 | 0 |
| SLC2A1 | NM_011400.2 | -1.895 | 0 |
| H2-AB1 | NM_207105.2 | -1.947 | 0 |
| H2-T23 | NM_010398.3 | -1.951 | 0 |
| EG630499 | NM_001081015.1 | -1.960 | 0 |
| FUS | NM_139149.1 | -2.000 | 0 |
| H2-D1 | NM_010380.3 | -2.002 | 0 |
| LOC547343 | NM_001034908.1 | -2.069 | 0 |
| H2-K1 | NM_001001892.2 | -2.125 | 0 |
| SGK1 | NM_011361.1 | -2.133 | 0 |
| NUDT11 | NM_021431.2 | -2.142 | 0 |
| GBP3 | NM_018734.2 | -2.151 | 0 |
| ASAH3L | NM_139306.1 | -2.275 | 0 |
| BAT5 | NM_178592.3 | -2.285 | 0 |
| CD74 | NM_010545.3 | -2.294 | 0 |
| GBP2 | NM_010260.1 | -2.341 | 0 |
| CD74 | NM_001042605.1 | -2.436 | 0 |
| IGTP | NM_018738.3 | -2.512 | 0 |
| EIF2S3Y | NM_012011.1 | -2.651 | 0 |
| H2-AB1 | NM_207105.2 | -3.020 | 0 |
| CSRP1 | NM_007791.4 | -3.617 | 0 |
