# Supplementary material for: Endogenous Methanol Regulates Mammalian Gene Activity
Source: PLoS One. 2014 Feb 27;9(2):e90239. doi: 10.1371/journal.pone.0090239 (PMC3937363; doi:10.1371/journal.pone.0090239)
Supplement: Table S4 — Oligonucleotides used for qPCR. (DOC) [file pone.0090239.s005.doc]

**Table S4. Oligonucleotides used for qPCR**

| **Gene** | **Forward Primer** | **Reverse Primer** | **PCR Size, Bp** | **Acquisition temperature,^o^C** |
| --- | --- | --- | --- | --- |
| m_RPL32 | GGCACCAGTCAGACCGATATG | CCTTCTCCGCACCCTGTTG | 80 | 56.0 |
| mADH | AGCCATTTCCACCCGTCAG | TTAGCAGCAAGCATATCCAAGG | 102 | 51.4 |
| mALDH1 | GAAGAAAGAAGGAGCCAAAC | CGCATCTCATCAGTCACG | 102 | 53.3 |
| mALDH2 | GACGCCGTCAGCAGGAAAA | CGCCAATCGGTACAACAGC | 189 | 62.9 |
| mAPOE | AGTGGGCAAACCTGATGG | ACCTGGCTGGATATGGATG | 127 | 53.9 |
| mCPLX2 | CGGGATGGGAGTGGGAAG | AAGGAAGCAGAGCAACAAGG | 111 | 55.4 |
| mCYP2D22 | TGCTGCTCCTCACAACTC | AACACTTCTCAATCCTAACTCC | 173 | 55.9 |
| mCYP2E1 | GAAGAAATTGACAGGGTTATTGG | GGAAGGGACGAGGTTGATG | 118 | 55.6 |
| mFIBCD1 | GTAGGTGGGAGTGTCATC | TAGAAGTGCTGGTTGTGG | 133 | 55.5 |
| mGSTO1 | ACGCTGATGGTCCTGAAG | GTGATGACAGATTCGGTGAC | 155 | 55.1 |
| mGSTP1 | TGCCACCATACACCATTG | TAACCACCTCCTCCTTCC | 102 | 54.6 |
| mMGST3 | ACGGTGGGAGGTGTTTAC | CAAAGAGGGCAAGAGAGC | 145 | 58.1 |
| mNDUFB6 | GAGCGATTCTGGGATAAC | TGACATAATAGTGAACAAACC | 130 | 51.7 |
| mNDUFC2 | GAGGTTGCTAAGGCTGAG | TTCCACGAGAGGTTTGAC | 186 | 55.8 |
| mRUSC2 | GGAAGGAGGGGAGGAACAC | TGACGACCAGACTATTTACAGC | 153 | 56.3 |
| mSERPINA3H | CCATCTCCACCGACTACAG | ACATCCAGCACAGCCTTG | 145 | 55.4 |
| mSNCA | CTATGAGCCTGAAGCCTAAGAATG | GATGGAAGACTTTGAAACACACTG | 147 | 54.4 |
| mSRXN1 | TCCACATCAGCACCACTACC | GGCACCAAGAGGCAGACC | 114 | 55.4 |
| mTESK1 | CCTCCCCTGACCCATCTC | TGACCTGCTGCTTGCTTG | 124 | 53.4 |
